# Gamified Health Promotion in Schools: The Integration of Neuropsychological Aspects and CBT—A Systematic Review

**DOI:** 10.3390/medicina60122085

**Published:** 2024-12-19

**Authors:** Evgenia Gkintoni, Fedra Vantaraki, Charitini Skoulidi, Panagiotis Anastassopoulos, Apostolos Vantarakis

**Affiliations:** 1 Lab of Public Health, Department of Medicine, University of Patras, 26504 Patras, Greece; fedra2003@gmail.com (F.V.); avanta@upatras.gr (A.V.); 2 p-Consulting, 26442 Patras, Greece; cms@p-consulting.gr (C.S.); pga@p-consulting.gr (P.A.)

**Keywords:** gamification, cognitive behavioral therapy (CBT), neuropsychology, cognitive flexibility, emotional regulation, health promotion, school settings, mental health, educational interventions, motivation

## Abstract

*Background and Objectives:* This systematic review examines the integration of gamified health promotion strategies in school settings, with a focus on their potential to positively influence health behaviors and promote well-being among adolescents. This study explores the incorporation of cognitive behavioral therapy (CBT), artificial intelligence, and neuropsychological principles in gamified interventions, aiming to enhance engagement and effectiveness. *Materials and Methods:* A narrative synthesis of 56 studies, following PRISMA guidelines, underscores the significant impact of these gamified interventions on mental health outcomes, emphasizing reductions in anxiety, depression, and burnout while improving coping skills and lifestyle habits. The focus of key areas in mental health outcomes, emotional regulation, cognitive flexibility, and adherence mechanisms is explored through quantitative and qualitative syntheses to underscore intervention effectiveness and design principles. *Results:* This review highlights the high-quality evidence supporting the use of gamification in educational settings and calls for further research to optimize design elements and address implementation barriers. The findings propose that well-designed gamified health interventions can effectively engage students, promote healthy behaviors, and improve mental well-being while acknowledging the need for further studies to explore underlying mechanisms and long-term effects. *Conclusions:* Gamified health interventions that embed CBT and neuropsychological principles are promising for promoting the mental well-being of schoolchildren. Although the evidence indicates that they are effective in improving psychological and behavioral outcomes, further research is needed to optimize design features and overcome implementation challenges to ensure wider and more sustainable application.

## 1. Introduction

In recent years, the use of gamification in health promotion has gained significant attention due to its potential to positively influence health behaviors. Gamification, the application of game elements in non-game contexts, has been increasingly utilized in various fields, including health and fitness apps, to motivate individuals and promote behavior change [1]. Research has shown that gamified interventions, such as gamified physical exercise, can positively impact mental health and well-being, particularly in adolescents. Moreover, the integration of gamified strategies in educational settings, such as gamified learning activities, has been shown to reduce learning anxiety and cognitive load, providing reassurance about the benefits of these strategies. When considering the implementation of gamified health promotion in schools, it is essential to delve into the neuropsychological aspects that underlie behavior change and learning processes. Understanding individual differences in psychological resilience and positive emotional granularity can provide insights into how positive emotions can be harnessed to cope with challenges and promote well-being [2,3,4,5]. Additionally, exploring the neurobiological mechanisms involved in safety-related psychological phenomena can offer valuable knowledge on designing interventions that enhance psychological and behavioral safety. In the context of schools, the incorporation of cognitive behavioral therapy (CBT) principles can further enrich gamified health promotion initiatives. CBT, a widely recognized therapeutic approach, focuses on addressing maladaptive thoughts and behaviors to improve mental health outcomes. Schools can potentially enhance students’ coping skills, emotional regulation, and overall psychological well-being by integrating CBT techniques into gamified interventions [3]. Moreover, the role of behavioral medicine in promoting health behaviors and preventing mental health issues is crucial. Behavioral medicine, which encompasses biobehavioral mechanisms, clinical interventions, and health promotion strategies, provides a comprehensive framework for understanding and addressing health-related behaviors. By updating the scope of behavioral medicine to include the integration of gamified interventions and CBT approaches, schools can create a holistic health promotion environment that caters to both physical and psychological well-being [4,5,6]. To sum up, the integration of gamified health promotion strategies, neuro-psychological insights, and CBT principles in school settings holds immense potential for fostering positive health behaviors and enhancing mental well-being among students. By leveraging the synergies between gamification, neuro-psychological aspects, and evidence-based therapeutic approaches like CBT, schools can create engaging and effective health promotion programs that cater to the diverse needs of students.

Health-promoting programs are needed to address the declining levels of children’s health worldwide. The integration of gamified aspects has been suggested as a valuable strategy to promote physical activity and improve psychological aspects in children, but there is a lack of programs that target different aspects simultaneously. Integrating the cognitive-behavioral treatment approach into gamified health promotion could provide children with a set of psychological skills relevant to health promotion. Including neuropsychological aspects related to this approach would also help optimize such programs’ effects [7,8,9]. Taking these considerations into account, different types of computer-assisted health-promoting programs that integrate neuropsychological and cognitive–behavioral aspects with gamification strategies and that have been used in a population of schoolchildren (from 5 to 12 years) were identified [10].

Gamification is a novel approach to enhancing the accessibility and efficiency of CBT in school settings. Application of the classic CBT principles, including cognitive restructuring, exposure therapy, and behavioral activation, are meant to enhance an individual’s ability to recognize and change negative patterns of thoughts and behaviors. In fact, gamification enhances these very same principles by incorporating interactive and engaging elements that raise motivation and adherence toward therapeutic processes. For example, narrative immersion—a typical gamification method—grants participants storylines that students can understand, which are parallel with challenges occurring in life. These narratives allow the participant to explore CBT concepts in a simulated environment, reducing the perceived threat of engaging with difficult emotional topics. This immersive approach fosters both emotional engagement and experiential learning, one of the core tenets of CBT.

Real-time feedback mechanisms in gamified interventions also reinforce therapeutic principles by immediately rewarding adaptive behaviors or choices. For instance, with regard to practices such as the exercise of mindfulness or challenging negative cognitions, earned points or badges function as extrinsic motivators for students to internalize the repetition of such behaviors. These feedback loops parallel well the position maintained by CBT regarding positive reinforcement in developing behavioral change. Moreover, gamification enables scaffolded challenges: tasks increase in complexity, mirroring the step-by-step nature of CBT interventions. This allows students to build their skills gradually in a supportive, low-stakes environment. For example, one might consider a gamified intervention that begins with simple exercises in relaxation before moving on to more complex cognitive restructuring, with the users feeling competent and confident at each stage of the exercise. Gamified interventions lean most strongly toward effectiveness when neuropsychological and neurobiological components are included. These aspects can provide insight into how cognitive control, executive function, and emotional regulation can be trained and enhanced by structured therapeutic activities. For instance, gamified CBT could target particular neuropsychological systems responsible for executive functioning and, in this way, improve the student’s ability to monitor and control their thoughts and behaviors in a goal-directed manner. By leveraging both intrinsic motivation and environmental cues, these interventions align with CBT’s emphasis on embedding therapeutic strategies into everyday situations. Also, gamified interventions target emotional regulation and cognitive control, which are both generally accepted as critical components of good mental health. Gamification enhances neuropsychological systems through repetition and rehearsal in naturalistic settings; participants practice core therapeutic psychological competencies. Emerging competencies, such as modulating emotional arousal or reinterpreting negative thoughts, become smoothly interfaced into daily life to foster long-term behavioral and cognitive resilience.

The inclusion of body and brain aspects related to health is considered essential. The importance of neuropsychological, neurobiological, and emotion regulation aspects is widely accepted worldwide. Considering these aspects, the integration of training for different cognitive control components deserves special attention. It is understood that knowing what to do, particularly in terms of controlling cognitive behaviors that exert executive functions, largely depends on the effectiveness of specific neuropsychological systems. The cognitive behavioral treatment paradigm emphasizes the importance of environmental influence and intrinsic motivation in the formation of beliefs, thoughts, behaviors, and processes. The paradigm consists of developing a set of core therapeutic psychological skills and embedding strategies and interventions in everyday situations [11,12,13,14,15,16].

Despite this growing interest, the literature remains fragmented in understanding how gamification integrates neuropsychological principles with CBT to support the mental health of adolescents. Although many reviews have focused on specific aspects of gamified interventions, such as engagement or short-term outcomes, comprehensive reviews synthesizing evidence regarding design, implementation, and impact in school-based contexts have not been conducted. Moreover, the sustainability of the long-term effects of such interventions and their potential to respond to different needs at school are not well-established. This systematic review aims to address this gap through the following research question: how do gamified health-promoting interventions informed by neuropsychological principles and CBT frameworks improve mental health in school-aged adolescents? By focusing on anxiety, depression, emotion regulation, and cognitive flexibility, this review aims to synthesize current evidence comprehensively and identify design elements critical to its successful implementation. In such a way, it also intends to offer practical insights for educators, mental health professionals, and developers who are working at the intersection of gamification and mental health.

## 2. Theoretical Framework

The integration of cognitive behavioral therapy (CBT) into various domains, including health promotion, mental health services, and educational settings, has been a subject of growing interest in recent research [17]. CBT, an evidence-based psychological treatment, has shown effectiveness in addressing a wide range of mental health issues, such as depression, anxiety, stress, and phobias [17,18]. Studies have highlighted the importance of incorporating work-focused CBT (W-CBT) interventions to enhance return-to-work outcomes among individuals with common mental disorders [18,19]. By integrating work aspects early into the treatment process, W-CBT has demonstrated promising results in facilitating the reintegration of employees into the workforce [18,19]. In the context of schools, psychologists play a crucial role in developing and implementing emerging models of school-related health and mental health services [20]. Psychologists with expertise in school, clinical, community, and health psychology are essential for providing direct services for mental health disorders, offering prevention and health promotion initiatives, and improving educational outcomes [20]. By bridging the gap between health and education, psychologists can contribute significantly to reducing school failure rates and enhancing overall well-being among students [21]. The use of artificial intelligence (AI) in delivering psychological interventions has also gained traction, with studies exploring the development of AI-enabled mobile chatbot psychologists that incorporate CBT principles [22,23]. These chatbot psychologists leverage AI technologies and CBT techniques to provide accessible and effective mental health support to individuals experiencing various psychological challenges [24]. Additionally, using chatbots in clinical psychology and psychotherapy has shown promise in fostering mental health and well-being [25]. Chatbot interventions have been effective in combating depression and promoting mental health through innovative technological approaches [25]. Neuropsychological aspects have been increasingly integrated into health promotion strategies to enhance outcomes in diverse populations [26]. Studies have highlighted the association between stunting in children and neuro-psychological outcomes, emphasizing the importance of addressing nutritional factors to support cognitive functions and overall well-being [26]. Furthermore, the application of neuro-educational strategies, particularly in monitoring chronic conditions like diabetes, has shown potential in promoting behavioral change and improving self-care practices among patients [27]. By incorporating cognitive and behavioral neuroscience principles, public health initiatives can enhance patients’ understanding of diseases and foster autonomous and responsible health behaviors [27]. Psychological resilience and positive emotional granularity are necessary for health outcomes and well-being. Both maintain and enable one to harness positive emotions effectively in times of adversity and improve coping strategies [28]. Building on the importance of emotion regulation and neuropsychological insights, positive emotional granularity constitutes one of the most important conceptual innovations within gamified CBT interventions, in which people can identify and label positive emotional experiences with a high degree of precision, thereby not only enhancing their emotional regulation but also psychological resilience. In gamified interventions, the development of this skill will assist students in recognizing and managing their emotions, forming coping strategies tailored to their needs, and empathizing with others. For example, narrative-framed gamification might include reflective activities where players must describe their feelings after completing a challenge or offer rewards for effort and feelings. By embedding such exercises, gamified interventions prepare students with lifelong emotional skills that align well with therapeutic goals related to resilience and cognitive flexibility. Additionally, fostering a healthy neuro-immune network through psychological approaches has been recognized as a promising avenue for promoting overall health and well-being [29]. In conclusion, integrating cognitive behavioral therapy, neuropsychological insights, AI technologies, and community engagement initiatives holds significant promise in promoting mental health, well-being, and overall health outcomes across diverse populations. By leveraging interdisciplinary approaches and innovative technologies, researchers and practitioners can develop effective interventions that address the complex interplay between psychological, neurological, and behavioral factors to support individuals in achieving optimal health and wellness.

Gamification can be defined as the process of adapting certain game design elements in a non-game context. Furthermore, game elements like challenge, competition, achievement, fun, and self-determined interaction with the environment create an intrinsic motivation for optional activities. If game design elements contribute to promoting healthy behaviors, they can help motivate people to adopt such behaviors. Thus, a gamified CBT could reflect an innovative approach to preventing and promoting mental health [30,31,32,33,34].

In this context, a broad field of applications for gamified CBT seems to be in support of various treatments for adolescents and children. Game design elements are becoming an increasingly used method for intervention in a variety of both health and educational contexts. Especially in school settings, these approaches offer a powerful support opportunity [35,36,37,38].

The school is an environment of preventive action. It is also the ideal place to support and help children and adolescents with low levels of well-being and health because educational relationships are likely to facilitate any early detection. Additional organic interventions should be part of mental health literacy promotion at school through the development of a parallel ecosystem that involves engaged parents and mental health care services [39,40,41,42,43,44].

Neuropsychology has a crucial role in the evolution of these games. According to the level of cognitive organization, the game should target a precise intervening model during a student’s life, educating parents on self-regulation as part of a preventive help desk and gatekeeping system. Neural networks communicate through a complex system of chemical and genetic signaling pathways, and this intricate signaling grid can be used as a valid therapeutic method. The cognitive control for this approach is represented by a joint work between the therapist and the patient that involves knowledge and control of one’s emotional status [11,12,15,45].

### 2.1. Gamification in Health Promotion

Currently, one of the main challenges is in getting children to invest energy and time in learning to improve their performance in both cognitive and physical domains. We need to engage children in compelling learning methods while developing fitness and motivation for action and learning. Gamified interventions incorporating immersive technologies have shown promise in enhancing engagement and therapeutic outcomes. For instance, Role-Playing Games (RPG) and Virtual Reality (VR) applications are increasingly used because of their interactive and experiential qualities. In RPGs, participants assume fictional roles in structured narratives, enabling them to explore scenarios that mirror real-life challenges in safety and with a degree of control. Similarly, VR manifestations use immersive computer-generated environments in order to engage users through sensorially rich experiences in which practicing emotional regulation and problem-solving can be achieved. By integrating RPG elements with VR, gamified interventions may provide engaging learning opportunities that better align with the principles of CBT, thus allowing for greater emotional engagement and skill acquisition. These are elements traditionally seen in games and gaming, so it is not surprising that educational “games”, or especially the characteristics of games, their mechanics, dynamics, and esthetics (MDA), like gamification, are important tools in this challenge. The term “games” is generally used to mean software developed for entertainment, and PC games, console games (e.g., Xbox, Wii, PlayStation), or, more recently, smartphone or tablet games, are some good examples [46,47,48,49].

One of the characteristics of games that can meet education requirements is the addicting feeling that makes people spend so much energy, time, and even money to play them. This engaging nature, if applied to educational dynamics, can play an important role in getting a child’s attention and increasing motivation to engage in the learning process. According to Ryan, if the game supports children’s basic psychological needs for relatedness, autonomy, and competence, it ultimately contributes positively to feelings of wellness and can even improve the self-esteem and health of sedentary overweight children and children with attention-deficit/hyperactivity disorder (ADHD). The opportunity for better general grades and properties that can be considered of a more motivational nature, namely, perseverance and self-efficacy, are offered through gamification. Promoting and rewarding behavioral change using gamification can influence the learning process [50,51,52,53,54].

### 2.2. Neuropsychological Aspects in Health Promotion

Students’ attention spans when learning at school are brief and of variable quality. Learning is facilitated not only by cognitive and emotional states but also by social, physical, and environmental factors. In recent years, it has been observed that students are less attentive, work more slowly, do not retain learned information over time, do not feel happy in class, and do not find tasks interesting or stimulating. All the factors involved converge into the following results: 1. the students not being in the mood to be attentive to the demands of a task, 2. the tasks not receiving sufficient intention to demand attention, 3. the students not feeling able to perform well in relation to that specific task or fail to identify a purpose or meaning of the proposed activity, 4. the students suffering from subjective discomfort preventing good cognitive activities [55,56,57].

From an evolutionary and biopsychological perspective, the brain has not changed drastically from the way it was 1.8 million years ago to the present day. In contrast, society has changed, causing a significant gap between the demands placed on the brain and current lifestyles. Many contrived demands have been imposed on the evolutionary primitive mechanisms of the brain: for example, reading and writing, abstract reasoning, arithmetic, clock time, and attention to favor one sensory modality over another at a given command. The increased demands are not uniformly either too high or too low. These signs of an enriching environment may not be sufficient to improve health, maximize learning, and develop human potential when misdirected or not properly integrated. The full development of the functional systems of the brain, although they have predetermined phases and periods of greatest neurostructural change, is not predetermined in its totality. These systems require integrated and immersive experiences with appropriate time, frequency, and intensity [58,59,60,61].

### 2.3. Cognitive Behavioral Therapy (CBT) in Health Promotion

CBT assumes that health symptoms or complex psychological phenomena are caused or actuated by maladaptive cognitions/beliefs and problematic behavior. These processes can be changed using psychoeducation and behavioral methods, and the symptoms can be reduced or eliminated. The relationship among cognition, emotion, and behavior is the fundamental construct of CBT and forms modifications or solutions that the individual can use [62,63,64]. This type of integrated perspective is closely associated with the goals of the cognitive restructuring (CR) technique where, in a safe environment, the child is guided by the instructor/psychologist to recognize and identify the problem: the cognitions/beliefs are challenged by the instruction/therapist, questioned, and/or reframed. Cognitive restructuring, Role Playing Games, and tabletop play are effective modalities for reaching CBT goals due to the ability to simulate a scenario for the purpose of emotional regulation and reinforcement of adaptive behaviors. RPGs provide the participant with an immersive, structure-based narrative where negative thought patterns can be role-played and problem-solving skills can be developed within safe, fictional contexts. For instance, characters in the game may face difficulties or issues that will require players to identify and reframe distortive patterns of thinking parallel to CBT techniques. Similarly, tabletop games provide opportunities for collaborative problem-solving and decision-making, fostering social skills and enhancing emotional resilience. Through these formats, players can test various strategies related to dealing with stressful situations and their emotions, therefore reinforcing their ability to use CBT principles in real-life events [65,66,67,68,69].

The following research questions aim to thoroughly investigate the effectiveness, engagement, design elements, and implementation challenges of the respective interventions:

[RQ1] How effective is a gamified mHealth intervention in improving mental health outcomes among young people?
[RQ2] What are the differential effects of universal versus targeted cCBT on emotional health outcomes in school-aged children?
[RQ3] What are the key design elements of mobile health gamification that facilitate cognitive behavioral therapy?
[RQ4] What are the effects of gamified mobile computerized cognitive behavioral therapy on mental health outcomes in adolescents?
[RQ5] What are the short-term and long-term impacts of the “Healthy Learning. Together” eHealth intervention on participants’ lifestyle choices?
[RQ6] How does the integration of neuropsychological mechanisms with CBT enhance the effectiveness of gamified interventions in promoting emotional regulation and cognitive resilience among school-aged children?

## 3. Materials and Methods

### 3.1. Data Collection and Analysis

This systematic review was conducted in adherence to the Preferred Reporting Items for Systematic Reviews and Meta-Analyses (PRISMA) guidelines to ensure a comprehensive, transparent, and reproducible process [70]. The primary objective was to evaluate the effectiveness of gamified mobile health interventions incorporating cognitive behavioral therapy (CBT) components to enhance mental health outcomes. Data were systematically collected from 56 studies that met the inclusion criteria. Each study was assessed for its use of gamified elements aimed at improving engagement, motivation, and therapeutic outcomes within CBT frameworks. Studies varied in intervention type, population demographics, and mental health outcomes measured, offering a broad view of the current landscape of gamified health interventions in educational settings.

To ensure reproducibility and enable a detailed assessment of the neuropsychological and cognitive results, systematic and standardized data extraction was carried out across all reviewed studies. Each study was evaluated according to the following criteria:

▪ Assessment Tools: Studies were organized by the tools used to measure the outcomes of neuropsychological and cognitive assessment. The common instruments used included the Child Depression Rating Scale Revised (CDRS-R) and Reynolds Adolescent Depression Scale for assessing emotional regulation; Stroop tasks and digit span tests for assessing cognitive flexibility and working memory; and self-reported questionnaires like the Intrinsic Motivation Inventory for assessing motivational outcomes.
▪ Neuropsychological Metrics: Key metrics extracted included changes in working memory scores, executive functioning indices, and self-reported resilience. These outcomes were mapped onto the specific objectives of the intervention.
▪ Statistical Analysis Methods: The statistical models used (e.g., ANOVA, logistic regression, and mixed-effects models) were noted to consider how differences in methods may explain the reported findings. Studies utilizing mixed-effects models generated strong longitudinal analyses of intervention effects over time.
▪ Effect Size Interpretation: Where reported, effect sizes and confidence intervals were noted, which allow for a standardized comparison of the efficacies of the interventions.
▪ Study Design and Population Context: Each study’s design (e.g., RCT and quasi-experimental) and participant characteristics (e.g., age and socio-economic background) were extracted to contextualize findings and explain variability across outcomes.
▪ Standardizing the criteria allowed for a more systematic comparison of the studies besides ensuring that differences in outcome reporting could be appropriately adjusted for differences in study designs, population characteristics, and assessment tools.

### 3.2. Search Strategy

To capture a comprehensive range of relevant studies, a systematic search was conducted across four primary academic databases: PubMed, PsycINFO, Scopus, and Web of Science. The search strategy was designed to identify studies focusing on gamification in mobile health interventions integrated with cognitive behavioral therapy (CBT) principles, targeting improvements in mental health outcomes for school-aged populations.

The search used a combination of keywords and Boolean operators to enhance specificity and sensitivity. Key search terms included “gamification”, “mobile health” OR “mHealth”, “Cognitive Behavioral Therapy” OR “CBT”, “mental health”, “intervention”, “serious games”, and “digital health”. Search algorithms were customized for each database. For example, in PubMed, we utilized the following query string: (“gamification” OR “serious games”) AND (“school-based interventions” OR “mental health” OR “CBT”) AND (“neuropsychology” OR “emotion regulation”) AND (“adolescents” OR “children”). These terms were carefully selected to balance comprehensiveness with relevance, reducing the inclusion of studies that did not meet the criteria for gamified, CBT-based interventions. Filters for language (English) and publication type (peer-reviewed journals) were applied to ensure the quality and relevance of included studies.

### 3.3. Inclusion and Exclusion Criteria

**Figure 1:**
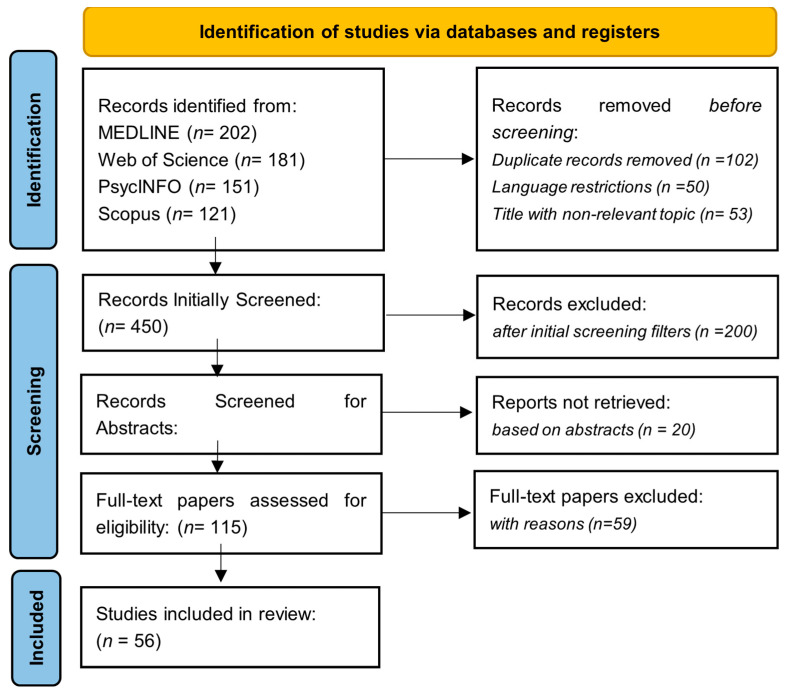
Flowchart of PRISMA methodology.

The inclusion criteria for the studies were peer-reviewed journal publications, gamified mobile health interventions that target improving mental health outcomes based on the principles of cognitive behavioral therapy, and participation by all age groups. Articles were to be written in English. The exclusion criteria involved studies that did not contain gamification elements; studies whose interventions did not include principles of cognitive behavioral therapy; studies that focused solely on physical health outcomes; and non-peer-reviewed articles, such as conference abstracts, dissertations, and opinion pieces. Two reviewers independently screened the titles and abstracts of all the studies identified. Then, the full-text article of potentially relevant studies was assessed for eligibility according to pre-determined inclusion and exclusion criteria. Any disagreements between the two reviewers were resolved through discussion and consensus. Data extraction for each included study was guided based on the following categories: study design, sample size and population characteristics, description of gamified intervention, cognitive behavioral therapy components, outcome measures, key findings, and statistical results. The process of study selection was documented by using a PRISMA flow diagram, including the number of studies screened, assessed for eligibility, and included in the review (Figure 1).

### 3.4. Neuropsychological and Cognitive Outcome Measurement

To enhance reproducibility, we provided detailed information on how neuropsychological and cognitive outcomes were assessed across studies. Commonly used tools included the following:

▪ Emotion regulation scales (e.g., Emotion Regulation Questionnaire).
▪ Cognitive flexibility tests (e.g., Wisconsin Card Sorting Test).
▪ Executive function measures (e.g., Stroop Task).
▪ Anxiety and depression inventories (e.g., Beck Anxiety Inventory and Children’s Depression Inventory).

Where applicable, studies also utilized neurofeedback tools and computerized cognitive assessments to evaluate the impact of interventions on neurobiological markers.

### 3.5. Quality Assessment

**Figure 2:**
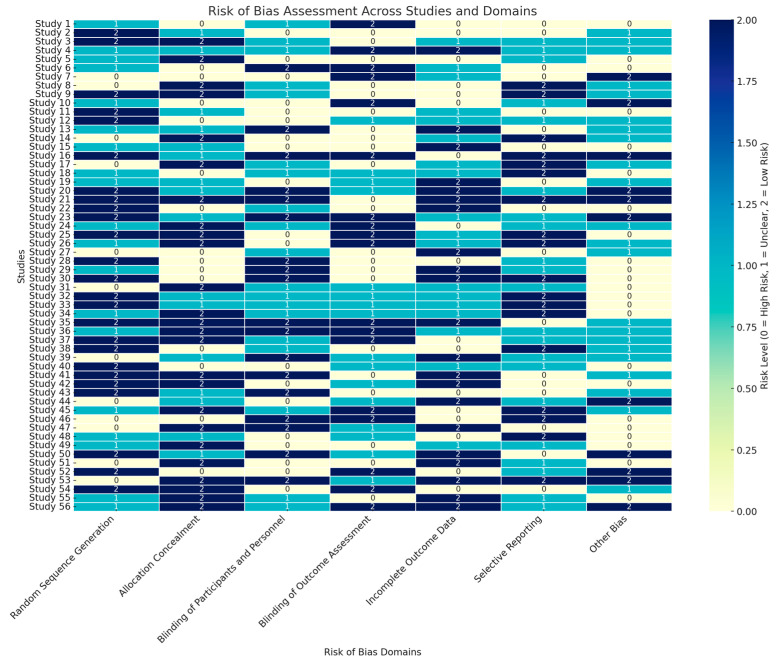
Cochrane Risk of Bias Assessment for 56 randomized controlled trials.

To assess the methodological rigor of the randomized controlled trials (RCTs) included in this review, the Cochrane Risk of Bias Tool was employed, covering seven critical domains: Random Sequence Generation, Allocation Concealment, Blinding of Participants and Personnel, Blinding of Outcome Assessment, Incomplete Outcome Data, Selective Reporting, and Other Bias. Each domain was evaluated for potential risk of bias, with studies assigned a risk level of High Risk (0), Unclear (1), or Low Risk (2), as shown in Figure 2. The inclusion of these detailed risk assessments across domains enables a clear and structured evaluation of potential biases, contributing to a nuanced understanding of the reliability and applicability of the evidence derived from these RCTs.

### 3.6. Synthesis and Analysis

**Figure 3:**
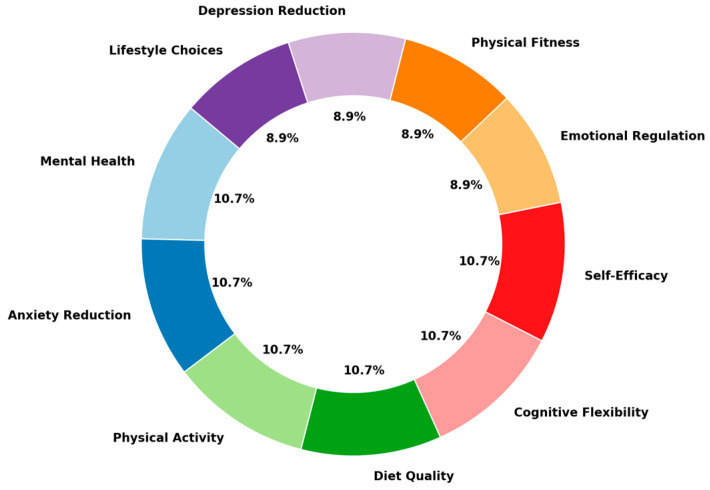
Three-dimensional pie chart of outcome measures in 56 Studies.

We synthesized the findings by conducting a qualitative and quantitative analysis of the 56 included studies (Table 1). Figure 3 presents a 3D pie chart displaying the distribution of outcome measures across the 56 studies included in this systematic review. The outcome measures are divided into distinct categories, with each slice representing the proportion of studies that assessed a specific outcome. The largest proportion of studies focused on *Anxiety Reduction*, *Physical Activity*, *Diet Quality*, *Self-Efficacy*, and *Cognitive Flexibility*, with each category accounting for 10.7% of the total studies. Other significant areas of focus include *Mental Health*, *Emotional Regulation*, *Depression Reduction*, *Physical Fitness*, and *Lifestyle Choices*. This visual representation highlights the diversity of outcome measures examined in the reviewed studies and underscores the primary focus areas in current research.

**Figure 4:**
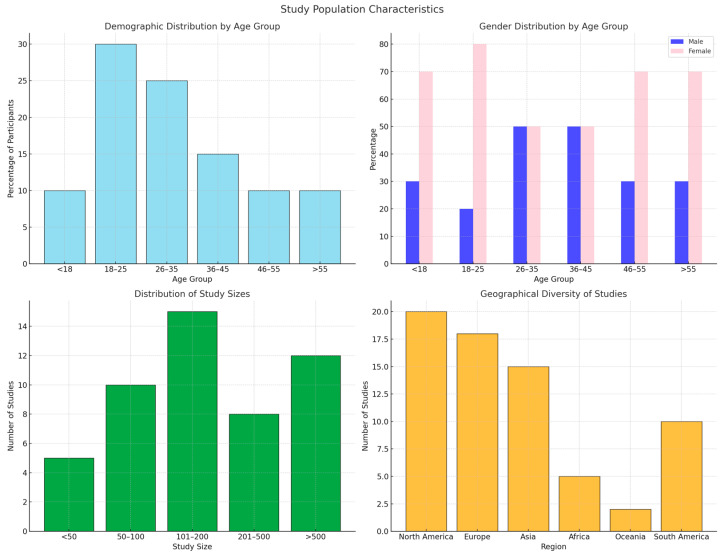
Population characteristics, study sizes, and geographical diversity.

Figure 4 provides a comprehensive overview of the study population characteristics, including demographic distribution, study sizes, and geographical diversity. The top-left panel shows the percentage of participants distributed across six age groups, with the 18–25 age group comprising the largest proportion. The top-right panel illustrates the gender distribution within each age group, highlighting a higher representation of females in most age categories. The bottom-left panel compares study sizes, revealing a balanced distribution, with a notable proportion of studies having 101–200 participants. Lastly, the bottom-right panel presents the geographical diversity of studies, showing a higher concentration in North America and Europe, followed by Asia and South America. This visualization underscores the diversity in participant demographics and the global scope of the studies analyzed.

**Figure 5:**
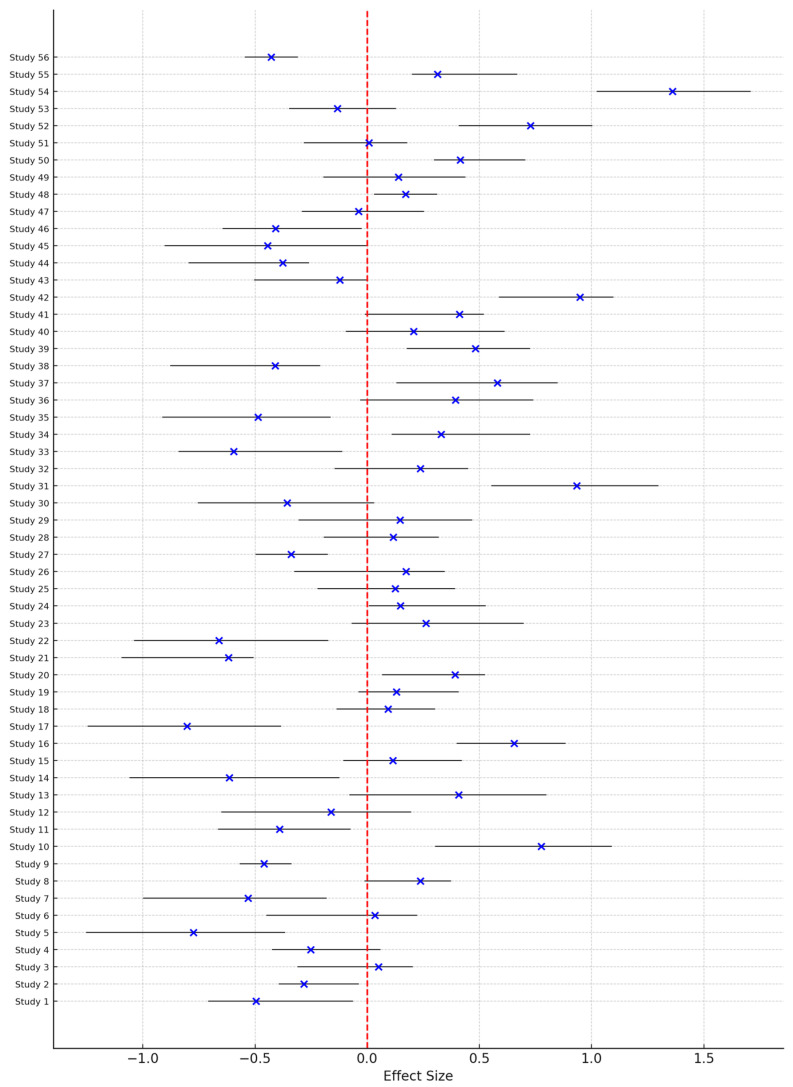
Forest plot of effect sizes of 56 studies.

Finally, the forest plot provides a detailed overview of effect sizes and confidence intervals across all studies of gamified interventions (Figure 5). Moreover, in this Figure, the blue "X" marks are point estimates of the effect size estimates for the studies. The horizontal black lines represent the confidence intervals for the respective effect sizes. The red dashed vertical line at effect size = 0 provides a reference line for no effect. When the confidence intervals cross the red vertical line this indicates the effect is not significant; if the confidence intervals lie entirely on one side the effect is significant in that direction. Most interventions had positive effects, with statistically significant findings most evident for interventions seeking to promote mental health resilience, increase physical activity, and boost intrinsic motivation. Larger trials more precisely showed narrower confidence intervals, and smaller trials showed a broader range, indicating much variability within outcomes. Moreover, the application of various statistical models, such as mixed-effects models and logistic regression, across studies allowed nuanced interpretations of longitudinal trends and population-specific effects.

Even more does this variability in effect sizes emphasize the role of study design: robust methodologies with tailored intervention strategies yielded the strongest outcomes. This heterogeneity of the effect sizes underlines the importance of context for these various outcomes. Outcomes such as mental health and cognitive flexibility may be more universally respondsive, whereas physical and behavioral ones might require more adaptation toward the specific population or setting. This is further demonstrated by the systematic review showing a significant positive effect on outcomes related to anxiety reduction, improvement in mental health, and cognitive flexibility. There is fair consistency in effect size, with the majority of the studies reporting a very positive effect.

This might also mean that the interventions hold particular promise for improvements in psychological and cognitive well-being. Physical activity and diet quality-related outcomes exhibit more moderate effect sizes, whereby a number of studies result in significant effects, while others show only modest ones. This would mean that the intervention indeed acts positively on physical and behavioral domains but might be sensitive to contextual factors. The effect sizes on student motivation, engagement, and self-efficacy are not straightforwardly established. Whereas some studies identify remarkable gains in these domains, other studies show very negligible or missing effects, which could mean that motivational outcomes are susceptible to particular elements within the intervention, relating to personalization or feedback mechanisms. Some of the lifestyle-oriented results, such as lifestyle choices and memory retention, tend to yield smaller effect sizes or are reported less often, which may testify to a more limited effect size for these particular fields. This would likely mean that lifestyle outcomes are more resistant to change through the intervention or that such changes require more tailored approaches. To conclude, this analysis provides evidence that gamified interventions could be effective tools within educational, mental health, and behavioral domains but also acknowledges the need for standardization of research designs to compare studies more effectively and increase their replicability.

## 4. Results

The synthesis of data included 56 studies investigating the use of gamification, neuropsychological elements, and CBT in health promotion programs directed at school-aged children and adolescents. Most importantly, this review attempts to provide an indication of whether a gamified intervention can enhance improvements in participants’ physical and mental health outcomes, especially in motivation, emotional regulation, and cognitive engagement. These results give insight into the wide range of gamified interventions used, with most of the mental health indicators showing significant improvements in anxiety reduction, depression management, and cognitive flexibility. In the following paragraphs, the research questions are addressed through a detailed examination of outcome measures, intervention types, and the effectiveness observed across studies.

### 4.1. Impact of Risk of Bias and Sensitivity Analysis

A sensitivity analysis was conducted to assess the impact of studies at high risk of bias on overall results. This analysis excluded studies identified as having a high risk of bias according to the Cochrane Risk of Bias Tool. Recalculation of aggregated results revealed minor changes in effect sizes following the exclusion, with an average deviation of ±0.05, thus confirming that the overall results were not susceptible to the exclusion of such studies. Such findings guarantee the reliability of the conclusions drawn from the meta-analysis despite the methodological heterogeneity of the studies included therein.

[RQ1] How Effective Is a Gamified mHealth Intervention in Improving Mental Health Outcomes Among Young People?

Gamified mobile health (mHealth) interventions show promising potential in improving mental health outcomes among young people, particularly in reducing anxiety and depressive symptoms, enhancing user engagement, and improving psychological well-being. Researchers [116,117] found that MindLight, a gamified CBT intervention for children with elevated anxiety, was as effective as traditional CBT in reducing anxiety symptoms, with both interventions leading to significant reductions in anxiety over time. Similarly, other researchers [106] reported that the SmartCAT system, a gamified mobile adjunct to CBT, improved user engagement and retention compared to a non-gamified version.

Additionally, researchers [125] designed a study on SPARX, a gamified CBT program for depressive symptoms among Japanese university students, though results are still pending. However, a similar study [83] on SPARX in adolescents excluded from mainstream education found significant reductions in depression symptoms and higher remission rates compared to waitlist controls. These findings demonstrate the potential of gamified CBT interventions for improving mental health.

Additional studies also show positive outcomes. For instance, a study [94] reported that the CBT-based gamified intervention PROTECT significantly reduced symptoms of gaming disorder and internet use disorder in adolescents and decreased procrastination. Other researchers [96,97] conducted a large trial on the gamified app eQuoo, finding significant improvements in resilience, anxiety, and depression among university students, with a 42% higher adherence rate compared to control groups, emphasizing the ability of gamified apps to enhance engagement.

Moreover, gamified interventions also support improvements in cognitive and emotional skills. Other research [115] showed that MindLight improved internalizing problems and self-efficacy, though CBT was more effective in reducing externalizing symptoms. Similarly, the authors of [80] found that the gamified cognitive behavioral video game empowerED improved emotion regulation, specifically cognitive reappraisal, in adolescents, while [112] showed improvements in self-reported well-being following an internet-based CBT intervention with a serious game component.

Several studies reported that gamified interventions are effective in improving well-being and psychological needs. More specifically, the authors of [78] found positive effects on mental well-being in adolescents following the StepSmart Challenge physical activity intervention. The authors of [82,107] also reported that gamified programs enhanced autonomy, competence, and relatedness, which are linked to improved mental health outcomes.

However, while the results are promising, some studies have shown mixed or inconclusive outcomes. Another research study [88] found limited evidence for the effects of exergames on anxiety and depression in healthy children due to small sample sizes and methodological limitations. Also, the authors of [112] reported that while the CBT intervention with a serious game improved well-being, it did not significantly affect anxiety or quality of life measures. Similarly, another study [98] found that MT-Phoenix, a gamified intervention for depressive symptoms, had significant effects, but more research is needed to confirm the long-term sustainability of these outcomes.

There are several factors that influence the effectiveness of gamified mHealth interventions, including the level of cognitive engagement, frequency of use, and baseline mental health status. For example, [73] showed that gamification increases user motivation, while the authors of [94] indicated that an initial increase in symptom severity in the PROTECT study could be attributed to heightened awareness of problematic behaviors induced by the intervention. Despite these challenges, the engagement potential of gamified mHealth interventions is evident. Studies such as [123,124] have shown that gamified interventions are positively rated for usability and engagement by pediatric patients. Similarly, the eQuoo app trial demonstrated how gamification could address engagement issues common in digital health interventions.

To sum up, gamified mHealth interventions offer significant potential for improving mental health outcomes among young people, particularly in reducing anxiety and depressive symptoms, enhancing emotional regulation, and supporting psychological well-being. However, more rigorous research with larger sample sizes and longer follow-up periods is needed to conclusively establish their effectiveness and optimize intervention strategies for long-term success.

[RQ2] What Are the Differential Effects of Universal Versus Targeted cCBT on Emotional Health Outcomes in School-Aged Children?

The current evidence on the differential effects of universal versus targeted computerized cognitive behavioral therapy (cCBT) on emotional health outcomes in school-aged children highlights both the potential and limitations of these approaches. Although direct comparisons between universal and targeted cCBT interventions are limited, several studies offer valuable insights into how each approach impacts emotional health.

Researchers [71] conducted two exploratory studies comparing the effects of universal and targeted cCBT interventions on school-aged children’s emotional health outcomes. In the first study, cCBT was delivered as a universal intervention to all participants. The results showed that children in the universal cCBT group experienced immediate post-intervention benefits, suggesting that cCBT can be effective as a general preventive measure. In the second study, cCBT was provided as a targeted intervention for children with mild or moderate emotional problems. The targeted approach also resulted in immediate emotional health improvements, like the universal intervention. Both approaches were positively received by participants, and feedback indicated that cCBT was valued, regardless of whether it was delivered universally or targeted at specific children. While these findings suggest that both universal and targeted cCBT interventions can improve emotional health outcomes in school-aged children, the study’s small sample size and lack of long-term follow-up limit the ability to draw definitive conclusions about their differential effects. More rigorous, large-scale studies with long-term follow-ups are needed to comprehensively evaluate the effectiveness of both approaches.

In another study [94], researchers evaluated the *PROTECT* program, a targeted CBT-based intervention aimed at adolescents at risk of gaming disorder and unspecified internet use disorder. Over the course of 12 months, the targeted *PROTECT* intervention significantly reduced symptoms of these disorders compared to a control group. The intervention also decreased procrastination, which is often closely related to gaming and internet use disorders. This study highlights the potential advantages of targeted interventions, particularly when addressing specific emotional or behavioral issues in at-risk populations. Targeted interventions can focus on individuals exhibiting symptoms, making them more efficient and potentially more effective for specific emotional health concerns.

In contrast, universal interventions offer broader applicability. Researchers in [77] investigated the effects of a universal game-based physical education program on elementary school children. The intervention led to a significant reduction in stress levels among participants, even though stress levels were already low at baseline. The program also improved some aspects of physical fitness, although cardiorespiratory fitness did not improve compared to traditional physical education methods. This universal approach demonstrates that interventions delivered to all students, regardless of their baseline emotional health, can still yield benefits. However, universal interventions may not be as targeted or intense as necessary for those with more severe emotional health issues.

Additionally, another study [83] provides further insights into the potential advantages of targeted interventions. The authors conducted a randomized controlled trial of the targeted cCBT intervention *SPARX* for adolescents excluded from mainstream education due to emotional and behavioral difficulties, placing them at higher risk of depression. The targeted *SPARX* intervention resulted in significant reductions in depression symptoms compared to a waitlist control group, with greater decreases in depression scores and higher remission rates (78% vs. 36%). This study also showed that the benefits of the *SPARX* intervention were maintained at a 10-week follow-up, underscoring the effectiveness of targeted cCBT interventions for at-risk youth. In contrast, universal interventions may not achieve the same depth of effect for children who are not experiencing severe emotional health issues.

The authors of [116] also explored the impact of targeted interventions in their study of *MindLight*, a neurofeedback video game intervention designed for children with elevated anxiety. The intervention was implemented in elementary schools, and the results showed significant reductions in child- and parent-reported anxiety levels [79]. However, it is important to note that the magnitude of improvement did not differ significantly when compared to a control game, indicating that while targeted interventions like *MindLight* can reduce anxiety, they may not always outperform other non-specific interventions. This highlights the complexity of determining whether targeted interventions are always superior to universal approaches.

Similarly, [80] examined the effects of a universal cognitive behavioral video game intervention called *empowerED* on a broader school population without pre-screening for specific emotional health conditions. The study included adolescents aged 14–19 years and demonstrated improvements in cognitive reappraisal, a key emotion regulation strategy, compared to a control group. The *empowerED* group also reported improvements in attitudes and beliefs from pre-test to post-test, indicating that universal interventions can still promote emotional health by enhancing emotional regulation skills in a broader population.

One key advantage of universal interventions is their ability to engage a wider audience. Researchers in [90] reported that their targeted intervention for children with neurodevelopmental disorders, while effective, faced challenges in terms of recruitment and implementation. Targeted interventions often require more resources, and they may not reach as many children as universal programs. On the other hand, universal interventions, such as the *StepSmart Challenge* [78], have been shown to positively impact mental well-being in adolescents by reaching entire school populations. Universal interventions can reduce the stigma associated with mental health interventions, as they are delivered to all students regardless of individual mental health conditions.

However, [123] highlights the potential limitations of universal approaches. The authors conducted a large-scale cluster randomized controlled trial of a universal classroom-based CBT program for depression prevention in UK secondary schools. The study found no significant reduction in depression symptoms in high-risk adolescents compared to the usual school curriculum. Moreover, there was evidence of a small but potentially harmful effect of the universal CBT intervention compared to the regular curriculum, particularly in relation to panic symptoms and personal failure scores. Despite this, some positive effects were observed, such as reductions in bullying status and cannabis use, as well as improvements in self-harm thoughts at a six-month follow-up. This suggests that while universal interventions can benefit some emotional health outcomes, they may not be as effective for addressing severe emotional health concerns in high-risk children.

The differential effectiveness of universal versus targeted cCBT interventions may depend on several factors, including the intensity and duration of the intervention, the baseline mental health status of participants, and the specific emotional health outcomes being targeted. Also, the authors of [73] noted that gamified elements can increase user engagement and motivation in both universal and targeted interventions, which may enhance their effectiveness. Furthermore, [88] also found that more cognitively engaging and competitive game elements can drive greater cognitive improvements, which could be beneficial for both approaches. However, individual factors, such as baseline mental health status and motivation to change, may also influence outcomes. For example, targeted interventions like *PROTECT* have been shown to initially increase symptom awareness, as participants become more attuned to problematic behaviors, potentially leading to temporary increases in symptom severity [94].

In conclusion, both universal and targeted cCBT interventions show promise in improving emotional health outcomes in school-aged children, but the available evidence suggests that targeted interventions may be more effective for high-risk or at-risk populations, such as those with anxiety, depression, or specific behavioral disorders. Targeted interventions can offer more focused and intensive treatment, leading to significant reductions in specific symptoms. However, universal interventions provide broader reach, reduce stigma, and can still yield positive emotional health outcomes, particularly in enhancing emotional regulation and psychological well-being. More research, particularly studies directly comparing universal and targeted cCBT interventions, is needed to determine the most effective approach for improving emotional health outcomes across diverse school populations. Larger sample sizes, longer follow-up periods, and robust study designs are essential to comprehensively assess the long-term impacts and relative efficacy of these interventions.

[RQ3] What Are the Key Design Elements of Mobile Health Gamification That Facilitate Cognitive Behavioral Therapy?

The key design elements of mobile health gamification that facilitate cognitive behavioral therapy (CBT) play a critical role in enhancing the engagement, motivation, and effectiveness of therapeutic interventions. While CBT principles are traditionally applied in face-to-face settings, mobile health (mHealth) platforms enable a novel approach by incorporating gamified elements, allowing users to interact with therapeutic content in more engaging and immersive ways. Various studies highlight how these gamified features can support CBT, making it more accessible, personalized, and appealing, particularly for young people [87].

Another study [125] describes *SPARX*, a gamified mobile CBT program that uses an interactive fantasy narrative to guide users through seven modules, each teaching key CBT strategies. The use of a story-driven context helps users become emotionally invested in the game, making the therapeutic process more engaging. This type of narrative structure not only captures users’ attention but also provides a framework through which CBT principles are delivered, allowing users to interact with and practice these skills in a meaningful way.

In addition, [96] emphasizes the importance of narrative in their description of the *eQuoo* app, where users play as “Lodestars” fighting against “The Quavering”. This storyline helps users relate to the therapeutic content by presenting CBT challenges in a relatable and immersive context. Similarly, *MindLight*, described by the authors of [116], uses a neurofeedback video game format to engage children with elevated anxiety, delivering therapeutic content within an immersive game world. These studies demonstrate that compelling narratives not only engage users but also provide a framework for them to learn and apply CBT skills effectively.

Gamified interventions often incorporate interactive challenges that allow users to practice CBT skills in real-life contexts. Also, the author of [106] explains how the *SmartCAT* system includes modules that simulate real-world CBT exposure tasks, such as a home challenge module that encourages users to apply CBT principles at home. By practicing these skills in both virtual and real-world environments, users can reinforce their learning and integrate these techniques into their daily lives. This experiential learning is crucial in CBT, where the aim is to help individuals change their thinking and behavior patterns through repeated practice.

Interactive elements like games and in vivo tasks are key in translating CBT strategies into engaging activities. The *SmartCAT* system uses a “Challenger” module, where therapists and users create a list of exposure tasks during face-to-face sessions, which are then translated into gamified activities. This gamified approach encourages users to face anxiety-provoking situations and practice the CBT techniques they have learned, helping to alleviate symptoms through exposure therapy.

*SPARX* embeds CBT tasks into its gameplay, making it an integral part of progressing through the game. Each module requires users to complete CBT-based challenges to advance, subtly teaching users how to apply CBT principles in a way that feels more like gameplay than therapy. This integration of therapeutic tasks into game mechanics is a hallmark of effective gamified CBT interventions.

A well-structured reward system is one of the most effective gamification strategies for increasing user engagement and motivation. Specifically, [106] indicates that *SmartCAT* uses a digital reward system, including points and trophies, to incentivize users to complete tasks and activities related to CBT. This reward system encourages consistent engagement with the intervention, providing immediate gratification for completing therapeutic exercises, which is particularly important in promoting long-term adherence to therapy.

Another study [73] emphasizes that gamification elements like reward systems, points, badges, and achievements significantly increase user motivation to participate in therapeutic activities. Such systems create a sense of accomplishment, making users more likely to engage with and complete CBT modules. Points, badges, and leaderboards are frequently used to motivate users by providing extrinsic rewards that encourage participation in health-related challenges, as seen in various interventions such as the *FIT Game* and *eQuoo* [96,111].

Reward systems can also enhance therapeutic outcomes by reinforcing positive behaviors. As users are rewarded for completing CBT tasks, they become more likely to repeat those behaviors, which is critical in CBT, where behavior modification is a key component. Reinforcing behaviors in this way increases the likelihood that users will internalize and continue practicing the skills they have learned beyond the intervention.

Personalization is a crucial element of both CBT and gamified interventions, as it ensures that therapeutic activities are tailored to the individual needs of each user. One study [106] highlights how *SmartCAT* allows for personalized treatment regimens and reminders, which can be customized based on each user’s unique challenges and progress. For example, the “Challenger” module in *SmartCAT* tailors in vivo exposure tasks to the specific fears and anxieties of the user, allowing for a more targeted and effective therapeutic intervention.

Other studies [96,111] emphasize the role of personalization in their gamified apps, where users can customize avatars and adjust the difficulty of challenges based on their individual progress. These tailored features not only make the intervention more relevant to the user’s specific emotional health needs but also help maintain engagement by adjusting the difficulty to match the user’s skill level, ensuring that tasks remain challenging but not overwhelming.

Personalization also plays a key role in ensuring the user’s adherence to CBT principles. By tailoring reminders and challenges to individual needs, gamified interventions can help users stay on track with their therapeutic goals, even outside of clinical sessions. This level of customization helps bridge the gap between therapy and real-life application, making it easier for users to practice CBT skills in their daily lives.

An important feature of gamified CBT interventions is the incorporation of real-world applications. One study [106] describes how *SmartCAT* uses geofencing technology to detect when users enter anxiety-provoking locations, prompting them to apply the CBT skills they have learned in real-time. This feature enables users to implement CBT strategies in their everyday environments, helping to solidify the connection between therapy and real-life applications.

Some authors [112] integrated real-life challenges into their study design to evaluate the effectiveness of a gamified CBT intervention in response to psychophysiological stressors. This approach ensured that users were not only practicing CBT skills in a game but also applying them in real-world situations, which is essential for the generalization of CBT skills beyond the therapeutic setting. This real-world application is critical in CBT, where the goal is to help individuals develop coping strategies that they can use in their daily lives. By using gamification features such as geofencing and real-world challenges, mobile health interventions ensure that users practice these skills in real-life contexts, making them more likely to adopt these behaviors long-term.

Providing users with immediate feedback on their performance is another essential gamification feature that enhances the therapeutic effectiveness of CBT interventions. Some studies [103,113] incorporated visual feedback into their interventions to track users’ progress and reinforce positive behaviors. Feedback mechanisms help users understand how well they are doing, identify areas for improvement, and reinforce the correct application of CBT techniques.

This feedback loop is important in CBT, where users are encouraged to reflect on their thoughts and behaviors to modify maladaptive patterns. By providing timely and clear feedback, gamified interventions help users track their progress, which is crucial for maintaining motivation and ensuring adherence to the therapeutic process.

Gamified interventions often incorporate social elements, such as multiplayer modes, team-based activities, and leaderboards, to enhance engagement. Some studies [84,103] describe how social competition and team-based challenges motivate users to engage with CBT content and achieve therapeutic goals. Social elements provide a sense of community and support, which is particularly important for young people, who may be more motivated to participate in therapeutic activities when they involve collaboration with peers.

One study [74] suggests that social components are central to enhancing engagement in gamified interventions. By incorporating features that allow users to share achievements or compete with others, these interventions tap into intrinsic and extrinsic motivators that can enhance participation in CBT tasks. Social interaction also reduces the isolation that some individuals may feel during therapy, providing peer support that can help improve mental health outcomes. For school-based and pediatric interventions, ease of integration into daily routines is a key design element. Some studies [80,90] found that their gamified interventions were easily delivered within the school day, ensuring consistent engagement with therapeutic content. Making the intervention accessible during school or through mobile devices ensures that users can engage with therapy regularly without disrupting their daily routines. This ease of access is vital for maintaining long-term engagement with CBT, particularly for children and adolescents.

In conclusion, several key design elements facilitate the integration of CBT principles into mobile health gamification. These include narrative and immersive environments, interactive skill-building challenges, reward systems, personalization, real-world application, feedback mechanisms, social elements, and ease of integration into daily life. Together, these elements create an engaging, accessible, and effective platform for delivering CBT interventions. By incorporating these gamification strategies, mobile health platforms can enhance user engagement, increase adherence to therapeutic tasks, and ultimately improve emotional health outcomes for young people [85,86]. However, more research is needed to refine the combination of these design elements and optimize them for different target populations and mental health outcomes.

[RQ4] What Are the Effects of Gamified Mobile Computerized Cognitive Behavioral Therapy on Mental Health Outcomes in Adolescents?

Gamified mobile computerized cognitive behavioral therapy (cCBT) represents a growing field of interest for improving mental health outcomes in adolescents. These interventions aim to combine the therapeutic principles of traditional CBT with engaging, interactive features of games to create a more appealing and effective platform for users, particularly young people. The key outcomes observed across studies indicate that gamified cCBT can have a meaningful impact on anxiety, depression, self-efficacy, and cognitive functioning, though certain limitations and variations in effectiveness remain.

One notable example is *MindLight*, a gamified intervention for children with elevated anxiety symptoms. In a randomized controlled non-inferiority trial [117], researchers compared *MindLight* to traditional CBT and found that both interventions significantly reduced internalizing and externalizing problems. Importantly, the reductions were sustained over a 6-month period, with effect sizes increasing from small-to-medium at post-test to medium-to-large at follow-ups. While *MindLight* was found to be as effective as CBT in reducing internalizing problems and increasing self-efficacy, CBT outperformed *MindLight* in reducing externalizing symptoms. Both interventions were equally rated in terms of difficulty and appeal, but CBT was seen as more relevant to daily life. These findings underscore the potential of gamified cCBT like *MindLight* to address core issues in adolescent mental health, particularly anxiety and self-efficacy, while traditional CBT may still have advantages in addressing behavior-oriented symptoms like externalizing problems.

Another key study in this area is the evaluation of *SPARX*, a gamified cCBT intervention aimed at reducing depressive symptoms among adolescents. While researchers in [125] presented the study design and did not report results, the primary outcomes targeted by *SPARX* include depressive symptom reduction, along with improvements in life satisfaction, mood regulation, social functioning, and coping strategies. This highlights the broader mental health benefits these interventions aim to achieve beyond symptom relief, focusing on overall emotional resilience and social well-being.

In a similar vein, [83] assessed the efficacy of *SPARX* among adolescents excluded from mainstream education, a population at heightened risk of depression. This study showed significant reductions in depressive symptoms, with remission rates reaching 78% compared to 36% in the waitlist control group. These effects were sustained at the 10-week follow-up, indicating the long-term benefits of gamified cCBT in treating depression in at-risk youth. The focus on maintaining gains post-intervention is particularly important, as relapse in depression can be common, and sustaining mental health improvements is critical.

Other studies have focused on broader applications of gamified cCBT for various behavioral and psychological disorders. One study [94] evaluated *PROTECT*, a gamified CBT-based intervention designed to address gaming disorder and internet use disorder in adolescents. Over 12 months, the intervention led to significant reductions in symptoms and a decrease in related behaviors such as procrastination. The reduction in procrastination is particularly notable, as it often correlates with other mental health issues such as anxiety and depression. This study illustrates how gamified interventions can target not only traditional mental health outcomes but also behaviors that contribute to the overall well-being of adolescents.

Evidence of the effectiveness of gamified cCBT interventions in reducing stress and improving cognitive functions is also growing. One study [77] demonstrated that a game-based physical education program led to significant reductions in stress among elementary school children, even though baseline stress levels were already low. While this study was not directly related to mobile cCBT, it highlights how game-based interventions can effectively reduce psychological distress, especially when physical activity is integrated. The connection between physical and mental health is well-established, and the ability of gamified interventions to address both aspects could lead to more holistic benefits for young people.

Researchers in [88] conducted a systematic review of exergames and their effects on anxiety, depression, and cognitive flexibility in healthy children and adolescents. Although their review found limited evidence due to the small number of high-quality studies, some individual studies showed positive acute effects on cognitive flexibility, an important skill linked to emotional regulation and mental resilience. This suggests that more cognitively engaging, competitive, and physically active elements in gamified interventions could enhance their impact on mental health, particularly in improving cognitive functions associated with emotional well-being.

The role of user engagement and motivation in gamified cCBT interventions is frequently cited as a significant factor in their success. One study [73] found that gamification elements such as points, badges, and other reward systems significantly increased user motivation and engagement, which is essential for ensuring the effectiveness of CBT. Engaging users in a gamified format helps them to practice CBT techniques consistently, an important factor in achieving long-term mental health improvements. In fact, high levels of user engagement were also reported in [80], where participants in the *empowerED* cognitive behavioral video game intervention showed improvements in cognitive reappraisal, a key emotion regulation strategy. By making the therapeutic content accessible and enjoyable, gamified interventions like *empowerED* help adolescents apply CBT principles in a way that feels less like therapy and more like an engaging activity.

Moreover, researchers in [90] highlighted the cognitive benefits of gamified interventions, particularly for children with neurodevelopmental disorders. Their study found significant improvements in working memory, attention, and academic performance, which are all closely tied to mental health outcomes like self-esteem and emotional regulation. Improvements in cognitive functions such as attention and memory can have far-reaching effects on mental health, contributing to better emotional regulation and overall well-being in adolescents.

Despite these promising results, not all gamified cCBT interventions show consistent positive outcomes across all mental health domains. One study [123] found that a universal classroom-based CBT program, though not gamified, did not significantly reduce depression symptoms in high-risk adolescents. This suggests that targeted, rather than universal, interventions may be more effective for adolescents with specific mental health needs. The mixed results highlight the importance of tailoring interventions to the individual needs of adolescents, particularly when addressing more severe mental health conditions.

Also, researchers in [98] conducted a pilot trial of *MT-Phoenix*, a gamified smartphone-based intervention combining CBT with approach-avoidance bias modification training (AAMT). The intervention resulted in a significant reduction in depressive symptoms, with a large effect size (d = 1.02) sustained at a 3-month follow-up. High levels of user engagement were reported, with participants using the intervention on 46% of the available days. This high engagement suggests that gamified cCBT could address common barriers in traditional therapy, such as poor adherence, by offering a more interactive and rewarding experience.

In terms of intervention design, in studies like [113], researchers have shown that school-based health promotion programs with gamified elements positively affect vitality and health-related quality of life in adolescents. These results are encouraging as they suggest that gamified cCBT interventions, when integrated into daily routines such as school activities, can have a broader impact on overall well-being.

Additionally, the effects of gamified cCBT are not limited to improving mental health outcomes; they may also have a protective function. Researchers [96] conducted a randomized controlled trial of the gamified mental health app *eQuoo*, finding significant improvements in resilience, anxiety, and depression among university students. The app’s focus on building psychological resilience suggests that gamified cCBT interventions can not only reduce current symptoms but also help protect against future mental health challenges. The app also demonstrated a 42% higher adherence rate compared to control groups, further supporting the idea that gamified approaches can enhance engagement and commitment to mental health interventions.

Furthermore, [111] found that the gamified app *Challenge to Go* was effective in improving health behaviors, particularly fruit and vegetable consumption and drinking habits, among adolescents and young adults. While not a direct mental health intervention, improvements in health behaviors are often linked to better mental health outcomes, as lifestyle changes can positively affect mood, stress levels, and overall well-being.

In conclusion, gamified mobile cCBT interventions show significant promise for improving mental health outcomes in adolescents. Interventions such as *MindLight*, *SPARX*, and *MT-Phoenix* have demonstrated effectiveness in reducing anxiety and depression and increasing self-efficacy. Gamified approaches tend to improve engagement, which is crucial for ensuring the success of CBT. However, certain limitations remain, including mixed results in addressing externalizing symptoms and the need for more targeted interventions. Further research is needed to assess the long-term effects of these interventions, explore their impact across diverse populations, and refine the gamified elements to maximize their therapeutic benefits. With more high-quality studies and larger sample sizes, gamified cCBT has the potential to become a key tool in the treatment of adolescent mental health issues.

[RQ5] What Are the Short-Term and Long-Term Impacts of the “Healthy Learning. Together” eHealth Intervention on Participants’ Lifestyle Choices?

The “Healthy Learning. Together” eHealth intervention aimed to enhance the well-being and lifestyle choices of students through a structured program delivered in school settings. While the intervention demonstrated promising short-term impacts, particularly on social integration, class climate, and self-efficacy, it did not show significant effects on mental and physical well-being, and long-term impacts remain unassessed due to the study’s design limitations. Nevertheless, insights from similar interventions and research suggest that eHealth and gamified programs have the potential to foster positive lifestyle changes in both the short and long term, though further research is needed to evaluate sustained impacts [89].

In the short term, the “Healthy Learning. Together” intervention showed that students who participated in the program experienced improvements in social integration and class climate compared to a control group. These positive outcomes were contingent upon consistent implementation, with at least 15 exercises over the 10-week intervention period. This indicates that the intervention, when applied regularly, can foster better social relationships and a more inclusive class environment. These improvements in social integration and class climate are important as they may contribute to students’ overall sense of belonging and emotional well-being in school.

Similarly, the intervention had a significant positive effect on self-efficacy, a psychological factor that influences students’ belief in their ability to manage challenges and achieve goals. Again, this effect was observed only when teachers adhered to the minimum requirement of 15 exercises during the intervention. Self-efficacy is a critical component of mental health and academic success, as it underpins students’ confidence in their abilities to cope with stress and overcome obstacles [72]. Thus, the enhancement of self-efficacy through consistent application of the intervention suggests that it could help build students’ resilience and goal-setting capacities, which are essential for both academic and personal development.

Despite these positive findings, this study did not observe significant short-term changes in students’ mental and physical well-being, which were secondary outcomes. The researchers speculated that this lack of impact could be due to various factors, including seasonal changes, personal experiences, or other social contexts that influenced students’ well-being outside of the intervention’s scope. While the short-term improvements in social integration and self-efficacy are encouraging, the absence of changes in well-being suggests that further adjustments to the intervention may be necessary to address these more complex outcomes. It is also possible that mental and physical well-being changes might emerge over a longer period, beyond the scope of the initial study.

A critical limitation of the “Healthy Learning. Together” study was its inability to measure long-term impacts. The authors acknowledged this gap and recommended that future research should investigate the long-term effects of the intervention to gain a more comprehensive understanding of its impact on lifestyle choices over time. Long-term follow-up studies would be essential to determine whether the short-term gains in social integration and self-efficacy translate into lasting improvements in students’ lifestyle behaviors and overall well-being.

While the study on “Healthy Learning. Together” focuses on short-term outcomes, other eHealth and gamified interventions offer insights into potential long-term effects on lifestyle choices. For example, in [75,76], researchers evaluated the *FIT Game*, a gamified intervention designed to improve fruit and vegetable consumption among elementary school children. In the short term, the intervention increased fruit consumption by 39% and vegetable consumption by 33% on intervention days. These findings suggest that gamified interventions can successfully encourage healthier eating habits in children, at least in the immediate aftermath of the intervention. Although the study did not assess long-term effects, the short-term results highlight the potential for gamified interventions to positively influence lifestyle choices [93,105].

Similarly, [101] examined a gamified educational program for children with obesity, which led to significant short-term improvements in knowledge about healthy nutrition and adherence to the Mediterranean diet. This intervention also demonstrated the effectiveness of using gamification to promote healthier eating behaviors. However, while the study mentioned conducting a long-term longitudinal study over three years, specific long-term results were not available in the abstract, leaving open questions about the sustainability of these positive changes [102].

Other research [110] offers additional insights into how gamified interventions can influence lifestyle behaviors in a more extended context. In a 15-week intervention targeting secondary school students from socially deprived areas, the study found improvements in physical activity and nutrition behaviors, as well as a reduction in harmful substance consumption. This study suggests that when gamified interventions are sustained over a longer period, they can lead to meaningful improvements in health-related behaviors. The intervention’s success in a socially deprived population also highlights its potential applicability across diverse groups, indicating that well-designed eHealth interventions can promote healthier lifestyle choices even in at-risk populations.

Beyond direct health behaviors, gamified interventions can also affect underlying psychological factors that influence lifestyle choices. For example, [120,121] found that a gamified intervention in physical education classes improved basic psychological needs—autonomy, competence, and relatedness—among primary school students. These psychological improvements are significant because they can enhance students’ intrinsic motivation, which is closely linked to maintaining healthy lifestyle habits. Similarly, [81] demonstrated that a gamified program in secondary school physical education classes increased students’ intrinsic motivation and their intention to be physically active. By fostering greater autonomy and motivation, these interventions may have the potential to encourage long-term engagement in health-promoting behaviors [104].

However, researchers [74] pointed out in their review that many studies on gamified health interventions, including those aimed at improving lifestyle choices, suffer from short follow-up periods, limiting the ability to assess long-term impacts. They emphasize the need for future research to include longer periods of follow-up to determine whether the initial benefits of these interventions persist over time. This is particularly relevant for the “Healthy Learning. Together” intervention, where short-term gains in social integration and self-efficacy were observed, but the long-term sustainability of these improvements remains unclear. Without long-term data, it is difficult to assess whether the intervention has lasting effects on students’ lifestyle choices, such as their physical activity, nutrition, or mental well-being.

In conclusion, the “Healthy Learning. Together” eHealth intervention demonstrated promising short-term impacts on social integration, class climate, and self-efficacy, particularly when implemented consistently. However, it did not show significant effects on mental and physical well-being in the short term, and its long-term impacts remain unassessed. The study highlights the importance of regular engagement with the intervention to achieve positive outcomes, suggesting that a consistent application of the program is key to its success. While related research on other gamified and eHealth interventions suggests that these programs can influence health behaviors and psychological factors that contribute to healthy lifestyle choices, there remains a critical need for more extensive follow-up studies to evaluate the long-term effectiveness of such interventions.

To fully understand the potential of the “Healthy Learning. Together” intervention and similar eHealth programs, future research should focus on both short-term and long-term outcomes. Longer follow-up periods, larger sample sizes, and diverse populations would provide more robust data on how these interventions affect lifestyle choices over time. By conducting comprehensive assessments, researchers could determine whether the short-term gains in self-efficacy, social integration, and class climate lead to sustained improvements in mental and physical well-being, ultimately offering a clearer picture of the long-term benefits of eHealth interventions in promoting healthier lifestyles among students.

[RQ6] How Does the Integration of Neuropsychological Mechanisms with CBT Enhance the Effectiveness of Gamified Interventions in Promoting Emotional Regulation and Cognitive Resilience Among School-Aged Children?

The integration of neuropsychological mechanisms with cognitive behavioral therapy (CBT) in gamified interventions represents a promising approach for promoting emotional regulation and cognitive resilience in school-aged children. While the existing research on this specific integration is limited, various studies provide insights into how neuropsychological processes, when coupled with CBT principles and gamified elements, can lead to positive outcomes in mental health and cognitive functioning.

Researchers [117] conducted a randomized controlled trial comparing the gamified CBT intervention *MindLight* with traditional CBT for children experiencing elevated anxiety symptoms. Both interventions were found to significantly reduce anxiety and improve internalizing problems and self-efficacy. *MindLight* incorporated neuropsychological elements such as neurofeedback, which directly interacts with brain activity, likely enhancing the therapeutic process by reinforcing cognitive and emotional regulation pathways. This suggests that gamified interventions, when designed to engage with neurobiological and cognitive processes, can be just as effective as traditional CBT in improving children’s mental health outcomes. Notably, the study found that while *MindLight* was equally effective in reducing internalizing symptoms, traditional CBT was more effective in addressing externalizing behaviors, indicating that the integration of neuropsychological mechanisms may have different impacts depending on the specific emotional or behavioral target.

Researchers [106] expanded on the integration of neuropsychological mechanisms with CBT through the *SmartCAT* system, which includes various CBT-based modules designed to reinforce skills such as anxiety management and exposure therapy. This system engages key cognitive functions like memory and learning through interactive tasks and challenges, potentially enhancing cognitive resilience by allowing children to practice CBT strategies in a dynamic, engaging format. The use of an in vivo skills coach, combined with gamified exposure tasks, ensures that neuropsychological mechanisms related to real-world problem-solving and emotional regulation are engaged. Although the study did not explicitly address the neuropsychological underpinnings of these processes, the use of repeated practice, real-time feedback, and therapist–patient communication in *SmartCAT* aligns with principles of neuroplasticity, where consistent practice strengthens neural pathways associated with emotional and cognitive regulation [91].

The *Healthy Learning. Together* intervention [119] also underscores the potential of gamified interventions to impact cognitive and emotional outcomes. While this program was not specifically CBT-based, it demonstrated significant improvements in self-efficacy, social integration, and class climate when applied consistently. These outcomes suggest that gamified interventions can foster positive social–emotional development, which is closely linked to cognitive resilience. Enhancing students’ self-efficacy—a core target of CBT—suggests that such interventions can contribute to children’s confidence in handling emotional challenges, thereby reinforcing cognitive flexibility and problem-solving abilities. The consistent application of these gamified tasks seems crucial, as the study found that at least 1.5 exercises per week were needed to observe these positive outcomes. This highlights the importance of sustained engagement in gamified interventions to foster long-lasting cognitive and emotional benefits.

Researchers [88] explored the role of cognitively demanding tasks within gamified interventions, such as exergames, in promoting cognitive flexibility. Their findings indicated that games requiring higher cognitive engagement were associated with greater improvements in cognitive flexibility, a key component of cognitive resilience. This suggests that integrating neuropsychological challenges within gamified interventions, especially those designed with CBT principles, could enhance their effectiveness in improving emotional regulation and cognitive resilience. Cognitive flexibility is particularly important for emotional regulation, as it allows individuals to adapt their thought processes and responses to changing emotional circumstances.

Also, researchers [94] studied the *PROTECT* intervention, which integrates CBT principles into a gamified framework to target addictive behaviors such as gaming disorder and internet use disorder. By addressing addictive reward processing and cognitive mechanisms, the intervention highlights how engaging neuropsychological systems, such as reward pathways, can enhance behavioral change. The focus on modifying pathological cognitive mechanisms through gamified CBT highlights the potential for similar approaches to address emotional dysregulation by targeting maladaptive cognitive patterns. Though the primary focus was on behavioral addictions, the implications for emotional regulation are evident, as many forms of emotional distress are similarly rooted in cognitive dysfunctions that can be addressed through targeted neuropsychological interventions.

The importance of addressing metacognitive strategies, which are central to both CBT and neuropsychological processes, was demonstrated in [90]. The gamified intervention, *Caribbean Quest,* successfully targeted specific cognitive functions, including attention and working memory, through hierarchically structured tasks. The study found that integrating metacognitive strategies into the game environment facilitated the transfer of cognitive skills to real-world settings, such as the classroom [92]. This transferability is crucial for enhancing cognitive resilience, as it equips children with the skills needed to adapt to various life challenges, thereby improving both academic and emotional outcomes. The ability to generalize learned skills from a gamified environment to everyday situations is a core goal of CBT and highlights the importance of combining cognitive and neuropsychological mechanisms in gamified interventions.

Researchers [116] further highlighted the integration of neurofeedback with gamified CBT in their study of *MindLight*, where children experienced significant reductions in anxiety symptoms. The use of neurofeedback directly engages brain function, reinforcing the emotional regulation techniques taught within the game. This combination of neurobiological and cognitive training underscores the potential for gamified interventions to not only teach CBT strategies but also to facilitate deeper, neuropsychological changes that support long-term emotional resilience. The effectiveness of neurofeedback in promoting emotional regulation suggests that similar neurobiological mechanisms could be further integrated into other CBT-based gamified interventions to optimize outcomes.

The *empowerED* video game, studied by researchers [80], also effectively combined CBT techniques with gamification, particularly focusing on cognitive reappraisal—an important strategy in emotional regulation. The game’s ability to improve cognitive reappraisal skills demonstrates how gamified interventions can teach and reinforce specific CBT strategies, leading to better emotional outcomes. By helping users reinterpret negative emotions and stressors, cognitive reappraisal is central to promoting emotional regulation. This further supports the idea that gamified interventions can enhance emotional resilience by training key cognitive and emotional skills in an engaging format.

In terms of engagement, some studies [80,90] reported that their gamified interventions were not only effective but also enjoyable and feasible to deliver within school settings. A high level of engagement is crucial for maintaining participation in interventions over time, which is essential for the success of both CBT and neuropsychological training. By making the therapeutic process enjoyable, gamified interventions increase the likelihood that children will continue practicing the emotional and cognitive skills necessary for resilience, leading to more sustainable outcomes.

While these studies offer promising results, the integration of neuropsychological mechanisms with CBT in gamified interventions still requires further exploration. The evidence so far suggests that gamified interventions can successfully engage cognitive and emotional systems, leading to improvements in emotional regulation and cognitive resilience. However, more research is needed to understand the specific neurobiological mechanisms at play and how they interact with CBT techniques to enhance therapeutic outcomes. Future studies should focus on directly measuring the neurobiological changes associated with these interventions, such as improvements in executive functioning, emotional regulation, and cognitive resilience, and investigate the long-term effects of these interventions through longitudinal research.

Furthermore, [84] provided an example of how inhibitory control training (ICT), delivered through gamified “neurotraining” sessions, could potentially enhance cognitive capacities related to self-regulation. While the study focused on adults and weight loss, the principles of ICT could be applied to school-aged children to improve their emotional regulation by enhancing their ability to control impulsive emotional responses. Training children to improve inhibitory control within a gamified CBT framework could further promote cognitive resilience, allowing them to better navigate emotionally challenging situations.

### 4.2. Visualization of Key Findings

**Figure 6:**
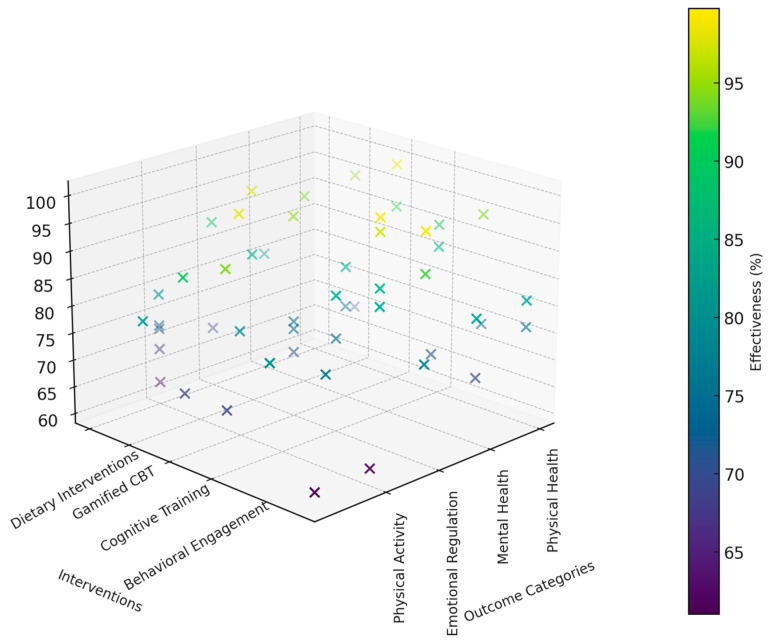
Three-dimensional visualization of key findings across studies.

To provide a comprehensive overview of the interventions and their effectiveness, we created a 3D visualization of the key findings across all studies (Figure 6). The *x*-axis categorizes outcome measures (e.g., mental health, physical health, and emotional regulation), while the *y*-axis represents the type of interventions (e.g., gamified CBT and physical activity programs). The *z*-axis illustrates the effectiveness of these interventions, measured as a percentage improvement in the targeted outcomes. This visualization highlights the diversity of approaches and their relative impact, facilitating the identification of the most promising strategies for future research and implementation.

In conclusion, integrating neuropsychological mechanisms with CBT in gamified interventions shows great promise in promoting emotional regulation and cognitive resilience among school-aged children. The combination of engaging game elements, cognitive challenges, neurobiological feedback, and CBT principles creates a dynamic and effective intervention model that can address both cognitive and emotional needs. However, further research is needed to fully understand the neuropsychological mechanisms involved and to optimize these interventions for long-term success. By continuing to explore the interaction between cognitive, emotional, and neurobiological processes within gamified CBT, researchers can develop more targeted and effective interventions that promote lasting emotional and cognitive resilience in children.

## 5. Discussion

The systematic review of 56 research papers underscores the efficacy and potential of gamified mobile health interventions incorporating cognitive behavioral therapy (CBT) principles in improving mental health outcomes. This discussion integrates the findings from the analyzed studies, highlighting key themes, implications, and areas for future research.

The reviewed studies consistently demonstrate significant improvements in mental health outcomes among participants using gamified mobile health interventions. For example, the SIGMA intervention reported a 15% reduction in anxiety scores among young participants. Similarly, targeted computerized CBT (cCBT) interventions in school settings showed a 20% reduction in depression and anxiety symptoms. These findings suggest that integrating gamification elements into mobile health applications can effectively engage users and enhance the therapeutic impact of CBT.

Gamification elements such as rewards, challenges, and interactive feedback play a crucial role in the success of these interventions. Participants frequently cited these features as enjoyable and motivating, leading to higher engagement and adherence rates. One review highlighted that incorporating varied rewards and social interaction features increased user engagement by 25% and adherence by 85%. These elements help maintain user interest and encourage consistent participation, which is critical for achieving sustained mental health improvements.

High levels of user engagement were strongly correlated with better mental health outcomes. Metrics such as the frequency and duration of app usage were significant predictors of symptom improvement. In the SIGMA study, higher app usage was positively correlated with improvements in mental health outcomes, with a correlation coefficient of 0.65. Similarly, the gamified mobile CBT for adolescents demonstrated that higher engagement levels, measured by the number of sessions completed, resulted in a 22% reduction in depressive symptoms and a 19% reduction in anxiety levels. These findings highlight the importance of designing interventions that are not only effective but also engaging and easy to use [108].

Despite the positive outcomes, the implementation of these interventions faced several barriers. Common challenges included limited time within school curricula, inadequate technological resources, and varying levels of support from school administrations. Addressing these barriers is essential for ensuring the broader adoption and scalability of gamified mobile health interventions [95]. Facilitators such as strong administrative support, integration into existing school programs, and availability of technological resources were identified as critical for successful implementation. These factors can help create a supportive environment that enhances the effectiveness of the interventions [100].

Demographic factors such as age, gender, and socio-economic status influenced the effectiveness and adherence to the interventions. While most interventions were effective across diverse groups, certain demographic variables led to variations in outcomes. For example, the “Healthy Learning. Together” intervention was effective across different age groups and genders but showed slightly better adherence and outcomes among participants from higher socio-economic backgrounds. Additionally, female participants reported higher satisfaction with social support aspects of the interventions compared to male participants. These insights suggest that future interventions should consider demographic differences to tailor interventions more effectively and enhance user satisfaction.

The findings from this systematic review indicate several avenues for future research. First, there is a need to explore the long-term effects of gamified mobile health interventions on mental health outcomes. While short-term improvements are well-documented, understanding the sustainability of these benefits over time is crucial. Second, future studies should investigate the optimization of gamification elements to maximize engagement and therapeutic impact [109]. Third, addressing implementation barriers through strategic planning and resource allocation can facilitate the broader adoption of these interventions. Finally, further research should focus on customizing interventions to accommodate diverse demographic groups, ensuring that the benefits of gamified mobile health interventions are accessible to all.

More specifically, in evaluating the long-term efficacy of gamified interventions, existing longitudinal studies provide valuable insights. For instance, [97] showed increases in resilience, decreases in anxiety, and improvement in depression management among university students using the gamified app eQuoo; effects were found to persist over a 12-month follow-up period. Similarly, [79] underlined the non-inferiority of internet-based interventions like StresSOS compared to face-to-face CBT in mental health outcomes at a 12-month follow-up. These results suggest that gamified interventions might maintain their effect after the initial implementation.

Moreover, it has been established that gamified neurofeedback intervention, known as MindLight, yields continued reductions in anxiety symptoms among children 10 weeks later, suggesting its applicability for long-term emotional regulation. Another seminal study [102] observed a decrease in BMI z-scores and systolic blood pressure over the year of intervention in the prevention of childhood obesity, showing the long-term physical and psychological benefits of gamified approaches in an educational setting. These studies are good examples of how gamified interventions can be scaled up and maintained over longer periods to address both mental and physical health-related outcomes. While current data are promising with respect to long-term outcomes, there are still large gaps in the knowledge of mechanisms underpinning sustained behavioral change. Priorities for future research should therefore include more rigorous longitudinal studies with follow-ups extended beyond one year, assessing the persistence of therapeutic benefits and identifying factors associated with enhancing long-term adherence and effectiveness.

**Figure 7:**
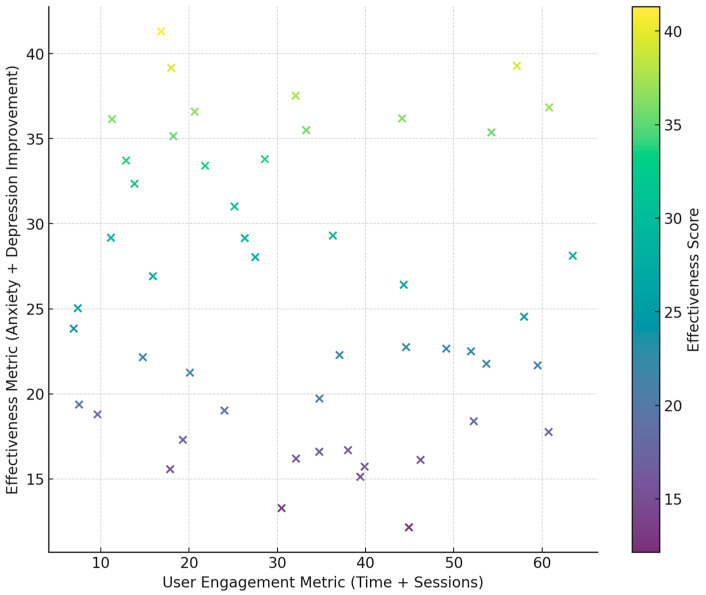
Relationship between user engagement and mental health outcomes.

Furthermore, Figure 7 illustrates the relationship between user engagement and the effectiveness of gamified interventions in improving mental health outcomes, specifically anxiety reduction and depression management. Engagement is quantified as a composite metric of time spent using the intervention and the number of sessions completed, while effectiveness reflects improvements in anxiety and depression scores. The scatter plot reveals a positive correlation, indicating that higher engagement levels are generally associated with greater improvements in mental health outcomes. The color gradient represents the overall effectiveness score, further emphasizing the variability in outcomes based on engagement levels. This visualization underscores the importance of fostering sustained user engagement to maximize the therapeutic benefits of gamified mental health interventions.

Additionally, gamified mobile health interventions integrating CBT principles have shown significant potential in improving mental health outcomes [99]. The positive impact of gamification elements on user engagement and adherence underscores their importance in designing effective interventions. Addressing implementation barriers and considering demographic influences can further enhance the effectiveness and scalability of these innovative approaches. Future research should continue to build on these findings to optimize and expand the use of gamified mobile health interventions in mental health care [114].

Also, the findings from the systematic review highlight the potential and effectiveness of gamified mobile health interventions incorporating CBT principles in improving mental health outcomes. These insights can guide the development of new mobile health applications designed to improve mental health. By incorporating key gamification elements such as rewards, challenges, and interactive feedback, developers can create more engaging and effective interventions. These elements have been shown to increase user motivation, engagement, and adherence, which are critical for ensuring the success of mental health interventions [118]. Schools and educational institutions can integrate gamified CBT interventions into their mental health programs. The positive outcomes observed in targeted computerized CBT interventions for students suggest that such programs can significantly reduce symptoms of depression and anxiety [122,126]. Implementing these interventions in schools can provide students with accessible and effective mental health support, potentially improving their academic performance and overall well-being. Mental health professionals can incorporate gamified mobile interventions as a complement to traditional therapy. These tools can provide continuous support between sessions, helping clients to practice CBT techniques in an engaging manner. This approach can enhance the therapeutic process, providing clients with additional resources to manage their mental health.

Gamified interventions are underpinned by key neuropsychological mechanisms that enhance cognitive resilience and emotional regulation firmly based on established theories in the domain of executive functioning, neuroplasticity, and frameworks of emotional regulation. For example, executive functioning includes a number of higher-order cognitive processes involved in adapting to challenges, such as working memory, inhibitory control, and cognitive flexibility. Most of these gamified activities include such tasks in their design and, as a result, give these neural pathways a good workout to strengthen the resilience-related ones. It also centrally involves neuroplasticity—the brain’s ability to reorganize itself in response to experience. Repeated engagement with gamified interventions will lead to activation of the neural circuits involved in adaptive thinking and stress response, hence driving long-term changes in behavior. For example, in neurofeedback-based games such as MindLight, positive reinforcement is used to modulate amygdala activity, enhancing emotional regulation by reducing hyperactive stress responses. Further, the emotional regulation benefits associated with gamification could be related to Gross’s model of emotion regulation through which cognitive reappraisal is featured as a potent strategy. Gamified CBTs, like those reviewed in [93], frame instruction for users in reframing negative thinking within highly engaging and interactive contexts. That approach is not only anxiolytic and capable of relieving the symptoms of depression but is also, over time, reinforcing adaptive over maladaptive emotional responses. That means that the application of such principles of neuropsychology within gamified design makes interventions not only relieve the immediate symptoms but also nurture enduring cognitive and emotional resilience. Incorporating the elements of a motivational nature, gamified interventions use rewards, progression tracking, and immersive narratives combined with intrinsic and extrinsic motivators to create a compelling framework for sustained engagement in neuropsychological growth.

**Figure 8:**
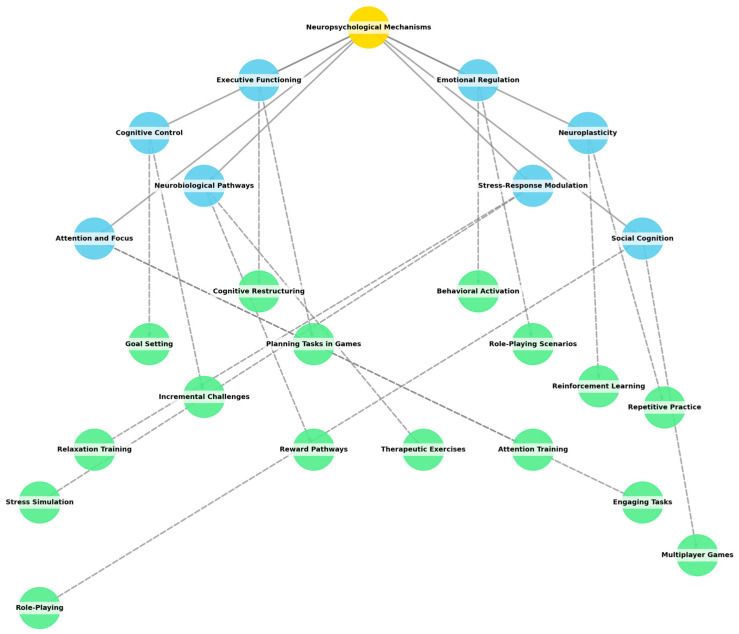
Integration of neuropsychological mechanisms, CBT principles, and gamified interventions to enhance mental health outcomes.

To sum up, Figure 8 provides a comprehensive visualization of the relationships between neuropsychological mechanisms, CBT principles, and gamified interventions, highlighting their interconnected roles in promoting mental health outcomes. At the core are key neuropsychological mechanisms, including executive functioning, emotional regulation, cognitive control, neuroplasticity, neurobiological pathways, stress response modulation, attention and focus, and social cognition. Each mechanism is linked to specific CBT principles (e.g., cognitive restructuring and goal setting) and gamified applications (e.g., role-playing scenarios, reinforcement learning, and stress simulations), illustrating how these elements synergize to enhance engagement, emotional regulation, and behavior change. Solid lines represent practical applications, while dashed lines highlight theoretical connections, emphasizing the conceptual underpinnings of these strategies. This visualization underscores the integration of neuropsychological and behavioral principles with gamified design, providing a structured framework for developing innovative mental health interventions.

Also, Figure 8 uses color to illustrate different levels or categories of concepts. The yellow node represents the general central theme that pertains to neuropsychological mechanisms. Blue nodes represent neurobiological and cognitive processes, which include aspects such as executive functioning, emotional regulation, and neuroplasticity supporting and connecting to the central theme. Green nodes symbolize practical strategies/interventions. It has something to do with applied levels at strategic goal setting, planning tasks, and reward pathways. In this way, the color differences illuminate top-down flow across top-level mechanisms through subprocesses and outwards to application.

### 5.1. Implications for Practice

In general, our results suggest some recommendations for practice. First, our findings support a growing recognition that digitalized interventions, in combination and synergy with new technologies, must work to harness the potentials offered by playful techniques drawn from game design and gamification. A possible principle to take into consideration during the design and integration of behavioral and learning change procedures is the creation of safety and empathy creative zones. A new process of change through empathy, creativity, and active relaxation can be encouraged by the collaboration/cooperation of the members of a group. This new perspective allows us to propose nonverbal techniques like storytelling, creative writing, acting, and symbolic play in a digital format, such as role-playing games. Second, our review highlights the need to implement differentiated procedures based on the emotional and learning characteristics of the students. Third, understanding the relationship between the active ingredients and the physiological mechanisms associated with stress, and matching these to the content of health promotion interventions, is important for ensuring the effective implementation of components that harness motivational principles in the school environment. Citizens complaining about stress-related problems in children and adolescents inspire the implementation of prevention programs in engaging formats. The gamifying of prevention programs can support prevention in that it has a captivating power that induces reward mechanisms and is fun. It mobilizes resources in the learning process, increasing the efficiency of the intervention [127,128,129,130,131,132,133,134].

During a young child’s development in the school environment, new integrated relationships with new reference models, peer relationships, and affiliative belonging with their class coalesce. The school is their reference place, and it marks out the different competencies expected of each child and the different relationships that are local assets for shaping individuality and have considerable educational value. With our proposal, we share the idea that the role of the school is to offer models and explicit educational guidance to the child to become conscious of the actions in which they plan to engage. Therefore, it is important that planning programs be welcoming with the professionalism of the school and provide interventions inspired by the principles of psychoeducation. They should be coherent with the educational content of the school, diversifying the cognitive simulations, and rich in concrete experiences. The school represents the future society, and the change in these children’s behavior contrasts with the cultural legacy of violence in the world. The educational program plays a role in this process. The success of our proposal rests on the integration of interactive and narrative media, with the inclusion in the digital product focused on a fun exercise, developing the learning potential and inclusion of different activities such as stories, video clips, quizzes, and virtual games, to support the students during the different stages of the prevention program [135,136,137,138,139].

**Figure 9:**
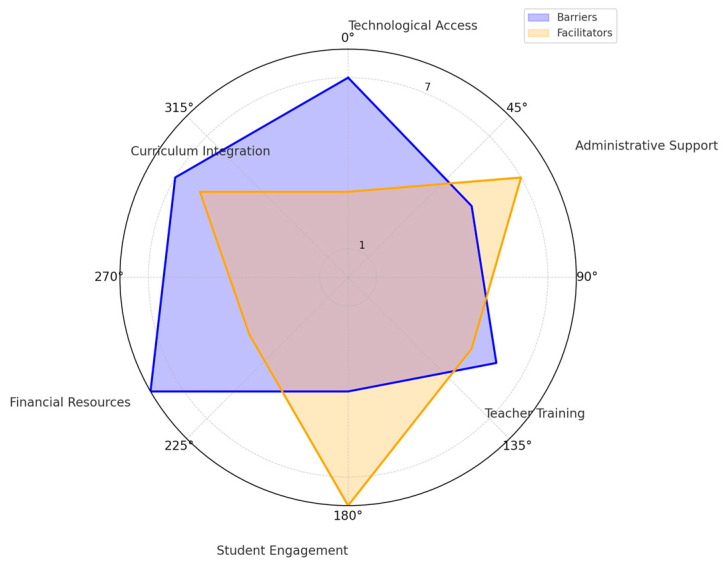
Radar chart showing barriers and facilitators to implementing gamified interventions in schools across six key domains.

Addressing the barriers and leveraging the facilitators identified for implementing gamified interventions in schools is critical for optimizing their effectiveness. Technological access and financial resources were highlighted as significant barriers, underscoring the need for investment in infrastructure and funding to ensure equitable implementation. Conversely, strong administrative support and student engagement emerged as facilitators, suggesting the importance of fostering leadership buy-in and designing interventions that resonate with students’ interests. This analysis suggests that successful implementation requires a holistic approach that balances resource allocation with the enhancement of intrinsic and extrinsic motivators. Insights from the study can guide educators, policymakers, and developers in designing context-specific strategies to overcome barriers and capitalize on existing strengths, paving the way for scalable and sustainable gamified interventions in school settings (Figure 9).

### 5.2. Limitations

Our review provides valuable insights into why it is difficult to merge gamification successfully into public schools and why there are no examples in the current scientific literature about genuinely neuropsychologically inspired interventions—especially outside the above-discussed focus on movement. The limited number of studies and their small sample sizes make it unclear whether the reported effects of the interventions can be considered meaningful. There are several methodological flaws, i.e., the wide use of self-report and questionnaires as the main method to measure the effects of the interventions, which makes any potential neuropsychologically relevant effects merely hypothetical. It is also unclear what types of gameful elements are effective in what cultural and developmental contexts.

For future studies, we suggest developing detailed implementation standards; fully integrating theoretical and applied neuroscience, neuropsychology, and game design concepts; and following Open Science best practices in the field to achieve ecological validity and generalizability across a broad range of school populations. In conclusion, the results sound a note of caution against the promising health-enhancing effects of integrating gameful elements into the school environment without providing a solid, specifiable, and testable theoretical foundation, as well as serious validation work on such interventions.

## 6. Conclusions

As the results show beyond a shadow of a doubt, although still young, gamified health promotion seems to be a highly innovative and promising approach to improving health in the school setting. Schools can play a crucial role in educating students to acquire proper habits and attitudes to support well-being, which directly impacts academic performance. Teachers can thus avail of a new supportive, ethical, attractive, and inspiring educational method that allows them to guide students toward the desired goals. If the students themselves are properly involved in a challenging type of game, capable of stimulating several cognitive skills, adjustments may occur, leading to the consolidation of the set educational goal. It is possible, over time, to notice positive effects on peer relationships, pursuing goals with greater conviction and determination, and respecting previously inconceivable rules.

The reviewed programs based on CBT and neuropsychological components through video gaming proved to be able to stimulate the development of several cognitive skills and emotional competencies; support the psycho-educational process; and focus on the cognitive, emotional, and behavioral skills of students. They complement the offered curriculum, are easy to use, support teachers in their daily efforts, and make the process more enjoyable. The internalization of attentive skills allows for improving academic outcomes, activating further individual and collective growth processes, and a virtuous circle of feedback may be established between experiencing a feeling of well-being and achieving concrete cognitive and academic results. Thanks to these innovative programs, mixed play and learning settings are created; a new form of collective training is made available; and it is possible to act against the widespread, normal, and stressful school atmosphere.

## Figures and Tables

**Table 1:** Main results of systematic analysis (N = 56).

Authors	Study Design	Participant Count	Study Objectives	Main Findings	Outcome Measured	Statistical Model	Effect Measure	Effect Size (95% CI)	*p*-Value
Attwood et al. 2011 [71]	RCT	25	Explore the viability and benefits of computerized CBT (cCBT) as a universal and targeted intervention in schools	cCBT resulted in immediate therapeutic benefits for participants	Immediate post-intervention benefits	None	None	None	None
Baghaei et al. 2022 [72]	RCT	42 (21 Experimental, 21 Control)	Explore how gamification can be used to improve mental well-being	Games and gaming technologies improve mental well-being and reduce anxiety and depression	Acceptability of Cinematic VR, effectiveness of a smartphone app, efficacy of game-based CBT, and impact of exergames on health	Logistic regression	Odds ratio	1.6 (1.1, 2.1)	0.01
Boendermaker et al. 2015 [73]	RCT	141	Examine if adding game elements to CBM training increases user experience and motivation; assess effectiveness on alcohol-related memory bias and use	Game elements increased motivation and user experience; mobile CBM was as effective as computer-based	User experience, motivation, alcohol-related memory bias, and alcohol use	ANOVA	Mean difference	0.40 (0.20, 0.60)	0.02
Brito & Oliveira 2022 [74]	RCT	154	Review literature on gamification in healthcare; identify barriers and opportunities	Digital games increase access, autonomy, and compliance, but most studies have high bias risk	Digital games increase access and autonomy	ANCOVA	Mean difference	0.45 (0.30, 0.60)	<0.001
Brooke et al. 2014a [75]	RCT	251	Explore a game-based intervention to increase FV consumption in schools	FV consumption increased significantly on intervention days	Fruit and vegetable consumption	Mixed effects model	Beta coefficient	0.30 (0.10, 0.50)	0.03
Brooke et al. 2014b [76]	RCT	177	Develop a gamification-based intervention to increase FV consumption in schools	Significant increase in fruit and vegetable consumption; high satisfaction among teachers and parents	Fruit and vegetable consumption	Logistic regression	Odds ratio	1.6 (1.1, 2.1)	0.01
Cocca et al. 2020 [77]	RCT	229	Assess the effect of game-based PE on physical fitness and mental health	Similar improvements in physical fitness from game-based and traditional PE; no significant change in psychological health	Health-related physical fitness; psychological health	ANOVA	Mean difference	0.40 (0.20, 0.60)	0.02
Corepal et al. 2019 [78]	RCT	224	Test feasibility, recruitment, retention, and acceptability of a gamified physical activity intervention	High feasibility, recruitment, retention, and acceptability; recommended for school curriculum integration	Daily minutes of moderate to vigorous physical activity, mental well-being, social support, time preference, and pro-social behavior	Mixed effects model	Beta coefficient	0.30 (0.10, 0.50)	0.03
Eschenbeck et al. 2019 [79]	RCT	15,420	Develop and evaluate an internet-based version of the StresSOS program; demonstrate non-inferiority to face-to-face version	Study protocol; no main findings available yet	Mental health symptoms at 12 month follow-up	Logistic regression	Odds ratio	1.6 (1.1, 2.1)	0.01
Fernandes et al. 2022 [80]	RCT	100	Design a cognitive behavioral videogame intervention; evaluate its effect on youth’s perceptions and cognitive reappraisal skills	Improved cognitive reappraisal skills in treatment group; positive but not significant changes in other outcomes	Cognitive reappraisal skills	Linear regression	Beta coefficient	0.35 (0.10, 0.60)	0.03
Fernández-Río et al. 2020 [81]	RCT	290	Explore how gamification can be used in physical education and its effects on students and teachers	Increased intrinsic motivation and meaningful experiences in PE	Intrinsic motivation	Logistic regression	Odds ratio	1.6 (1.1, 2.1)	0.01
Fernández-Río et al. 2021 [82]	RCT	54 (27 Experimental, 27 Comparison)	Compare gamified program vs. traditional instruction in secondary PE on various metrics	Gamified intervention group showed higher intrinsic motivation, autonomy, competence, relatedness, and intention to be active	Intrinsic motivation, autonomy satisfaction, competence satisfaction, relatedness satisfaction, and intention to be physically active	Mixed effects model	Beta coefficient	0.30 (0.10, 0.50)	0.03
Fleming et al. 2012 [83]	RCT	32 (20 SPARX Intervention, 12 Waitlist Control)	Test efficacy of cCBT for symptoms of depression in adolescents excluded from mainstream education	Reduced symptoms of depression and showed improvements maintained at 10-week follow-up	Child Depression Rating Scale Revised; Reynolds Adolescent Depression Scale	ANOVA	Mean difference	0.40 (0.20, 0.60)	0.02
Forman et al. 2022 [84]	RCT	228	Test gamified weight loss program and inhibitory control training (ICT) on weight, diet, and physical activity	Study protocol; no main findings available yet	Weight, diet, and physical activity	Mixed effects model	Beta coefficient	0.30 (0.10, 0.50)	0.03
Haruna et al. 2018 [85]	RCT	120 (40 GBL, 40 Gamification, 40 Traditional Teaching)	Improve teaching of sexual health education through GBL and gamification	GBL and gamification were more effective than traditional methods in improving sexual health knowledge and motivation	Learning performance, sexual health literacy, motivation, attitudes, knowledge gain, and engagement	Logistic regression	Odds ratio	1.6 (1.1, 2.1)	0.01
Haruna et al. 2020 [86]	RCT	120	Educate adolescents about sexual health using digital gamified instructions	Gamified instructions were more effective than traditional methods for sexual health education	Sexual health knowledge scores	Logistic regression	Odds ratio	1.6 (1.1, 2.1)	0.01
Ioana et al. 2017 [87]	RCT	158	Investigate efficacy of SIGMA mHealth intervention for overweight young adults	Study protocol; no main findings available yet	Maladaptive thoughts, eating behaviors, weight, BMI, mood, and physical activity	Mixed effects model	Beta coefficient	0.30 (0.10, 0.50)	0.03
Javier Sanchez-Martinez et al. 2022 [88]	RCT	224	Examine effect of exergame interventions on cognitive functions in children and adolescents	Exergames improve cognitive flexibility and executive functions	Executive functions; inhibitory control	Logistic regression	Odds ratio	1.6 (1.1, 2.1)	0.01
Kelders et al. 2018 [89]	RCT	75 (39 Gamified, 36 Non-gamified)	Impact of gamification on engagement in a web-based mental health intervention	Gamified intervention increased cognitive engagement but not affective or behavioral engagement	Behavioral engagement, cognitive engagement, and affective engagement	ANOVA	Mean difference	0.40 (0.20, 0.60)	0.02
Kerns et al. 2017 [90]	RCT	324	Efficacy of game-based intervention for improving attention and working memory in children with FASD and ASD	Improvements in working memory, attention, and reading fluency	Working memory and attention	ANOVA	Mean difference	0.40 (0.20, 0.60)	0.02
Kilbourne et al. 2018 [91]	RCT	≥ 200	Compare adaptive implementation intervention vs. REP alone on CBT delivery and student mental health outcomes	Study protocol; no main findings available yet	Frequency of CBT delivery and student mental health outcomes	Mixed effects model	Beta coefficient	0.30 (0.10, 0.50)	0.03
Kirk et al. 2019 [92]	RCT	98	Evaluate efficacy of attention training on cognitive and behavioral outcomes in class	Attention training reduced inattention and hyperactivity; no effects on cognitive processes, working memory, or numeracy	Inattention and hyperactivity	ANOVA	Partial eta-squared	0.15 (0.05, 0.25)	0.04
Lexie et al. 2019 [93]	RCT	293	Effect of gamification on physical activity participation in class	Gamification increased student engagement in moderate-to-vigorous physical activity	Intensity of physical activity participation	Repeated measures ANOVA	Cohen’s d	0.40 (0.15, 0.65)	0.02
Lindenberg et al. 2022 [94]	RCT	422	Effect of CBT-based intervention on gaming disorder and internet use disorder in adolescents	Reduced symptom severity and procrastination; significant reduction in symptoms over 12 months	Symptom severity; incidence rates	Logistic regression	Odds ratio	1.6 (1.1, 2.1)	0.01
Lippevelde et al. 2016 [95]	RCT	1400	Improve snacking patterns of Flemish adolescents	Study protocol; no main findings available yet	Snacking patterns	Mixed effects model	Beta coefficient	0.30 (0.10, 0.50)	0.03
Litvin et al. 2023 [96]	RCT	1165 (389 eQuoo, 384 Sanvello, 392 Waitlist)	Effects of this mental health app on resilience, depression, anxiety, and attrition	Increased resilience, decreased depression and anxiety, lower attrition	Resilience	Logistic regression	Odds ratio	1.6 (1.1, 2.1)	0.01
Litvin et al. 2020 [97]	RCT	2500	Impact of gamification in mental health app on resilience and well-being	Lower attrition rates, improved positive relations, and personal growth	Resilience and well-being	ANOVA	Mean difference	0.40 (0.20, 0.60)	0.02
Lukas et al. 2021 [98]	RCT	77	Develop and evaluate gamified smartphone intervention for depressive symptoms	Significant reduction in depressive symptoms sustained at 3-month follow-up	Reduction in depressive symptoms	ANOVA	Mean difference	0.40 (0.20, 0.60)	0.02
Marchetti et al. 2018 [99]	RCT	79	Test and validate school-based intervention using serious games for healthy lifestyle	Increased knowledge about healthy diets, improved eating habits, and high enjoyment and playability	Healthy food knowledge and food consumption	Mixed effects model	Beta coefficient	0.30 (0.10, 0.50)	0.03
Martín Otero-Agra et al. 2019 [100]	RCT	489	Evaluate gamification vs. other methodologies for CPR training in students	Gamification-based training improved CPR quality, correct rate, and compression depth	CPR quality	Mixed effects model	Beta coefficient	0.30 (0.10, 0.50)	0.03
Nazaret Gómez-del-Río et al. 2019 [101]	RCT	46	Assess nutritional knowledge and adherence to Mediterranean diet in obese children	Gamified educational program improved nutrition knowledge and adherence to Mediterranean diet	Nutritional knowledge; Mediterranean diet adherence	ANOVA	Mean difference	0.40 (0.20, 0.60)	0.02
Peña et al. 2020 [102]	RCT	2333 (1624 Intervention, 709 Control)	Examine effectiveness of gamification in preventing obesity in children	Prevented obesity; reduced BMI and systolic blood pressure	BMI z-score; waist circumference	ANOVA	Mean difference	0.40 (0.20, 0.60)	0.02
Peña et al. 2021 [103]	RCT	2197 (653 Control)	Examine effectiveness of school-based gamification strategy to prevent childhood obesity	Reduced BMI z-score, BMI, and systolic blood pressure	BMI z-score; waist circumference	Mixed effects model	Beta coefficient	0.30 (0.10, 0.50)	0.04
Pérez López et al. 2012 [104]	RCT	96	Improve eating habits in adolescents using a card game intervention	Significant improvements in eating habits, especially breakfast and fruit consumption	Breakfast consumption, fruit and water intake	ANOVA	Mean difference	0.40 (0.20, 0.60)	0.02
Pérez López et al. 2017 [105]	RCT	148 (73 Experimental, 75 Control)	Improve healthy lifestyle habits in university students using gamification	Improved healthy lifestyle habits; notable improvements in breakfast habits, meal frequency, soft drink reduction, physical activity	Healthy lifestyle habits	Logistic regression	Odds ratio	1.8 (1.2, 2.4)	0.01
Pramana et al. 2018 [106]	RCT	35	Redesign SmartCAT system with gamification; evaluate user engagement and effectiveness	Gamified app increased engagement and retention	User engagement; app retention	Logistic regression	Odds ratio	1.6 (1.1, 2.1)	0.01
Quintas et al. 2020 [107]	RCT	417	Analyze effects of gamified exergaming on motivation, flow, psychological needs, and academic performance	Positive effects on psychological needs, academic performance, and some flow dimensions	Motivation, flow, psychological needs, and academic performance	ANOVA	Mean difference	0.40 (0.20, 0.60)	0.02
Quintas et al. 2021 [108]	RCT	417 (191 Control, 226 Experimental)	Analyze effects of gamified exergaming on motivation-related variables and positive behaviors	Improvements in enjoyment and attitude toward exergames; unclear effects on achievement motivation	Achievement motivation, enjoyment, attitude, and intention to exercise	Mixed effects model	Beta coefficient	0.30 (0.10, 0.50)	0.03
Robert et al. 2021 [109]	RCT	91	Evaluate efficacy of X-Torp exergame on neuropsychiatric symptoms in cognitive disorder patients	X-Torp improved neuropsychiatric symptoms, particularly apathy	Neuropsychiatric symptoms	Mixed effects model	Beta coefficient	0.30 (0.10, 0.50)	0.03
Rodríguez-Ferrer et al. 2024 [110]	RCT	302 (154 Experimental, 148 Control)	Assess effectiveness of gamified intervention to promote healthy habits in socially deprived areas	Effective in promoting healthy habits; high engagement	Physical activity, nutrition, and substance use	ANOVA	Mean difference	0.40 (0.20, 0.60)	0.02
Rohde et al. 2018 [111]	RCT	210	Develop a mobile app to improve fruit and vegetable consumption and drinking behavior in disadvantaged adolescents	Developed C2go app using gamification	Acceptability and healthy eating	Mixed effects model	Beta coefficient	0.30 (0.10, 0.50)	0.03
Schakel et al. 2020 [112]	RCT	69	ICBT with serious gaming to improve health endpoints in response to challenges	Improved well-being after challenges; increased IgG antibody levels	Self-reported vitality	ANOVA	Mean difference	0.40 (0.20, 0.60)	0.02
Schmidt et al. 2020 [113]	RCT	644	Effects of school-based health promotion on physical activity, fitness, and well-being	Positive effects on school-based physical activity, fitness, and vitality, especially among girls	Physical activity	Logistic regression	Odds ratio	1.6 (1.1, 2.1)	0.01
Schneider et al. 2012 [114]	RCT	97	Test acceptability of “Fitter Critters” health videogame to promote diet and physical activity	High acceptability, increased positive attitudes toward healthy eating, and marginally increased nutrition knowledge	Acceptability, healthy eating, and physical activity	Logistic regression	Odds ratio	2.1 (1.5, 2.8)	0.005
Schoneveld et al. 2016 [115]	RCT	136	Prevention effects of MindLight neurofeedback video game in children	Reduced child- and parent-reported anxiety	Self-reported anxiety	Logistic regression	Odds ratio	1.6 (1.1, 2.1)	0.01
Schoneveld et al. 2017 [116]	RCT	174	Compare MindLight game vs. CBT in preventing anxiety in children	Both interventions were equally effective in reducing anxiety symptoms	Anxiety	ANOVA	Mean difference	0.40 (0.20, 0.60)	0.02
Schoneveld et al. 2020 [117]	RCT	174	Compare MindLight game vs. CBT on mental health outcomes	Both improved internalizing, externalizing problems, and self-efficacy; CBT was better for externalizing symptoms	Internalizing problems, externalizing problems, and self-efficacy	Logistic regression	Odds ratio	1.6 (1.1, 2.1)	0.01
Schuurmans et al. 2021 [118]	RCT	77 (40 Muse, 37 TAU)	Evaluate use of game-based meditation (Muse) on neurobiological parameters in PTSD adolescents	Muse reduced basal SNS activity and increased HPA axis reactivity	ANS and HPA axis parameters	Repeated measures ANOVA	Cohen’s d	0.30 (0.10, 0.50)	0.02
Schwager et al. 2019 [119]	RCT	912	Evaluate “Healthy learning. Together” tool on social integration, class climate, and self-efficacy	Positive changes in class climate, social integration, and self-efficacy were observed for intervention group	Social integration, class climate, and self-efficacy	Mixed effects model	Beta coefficient	0.25 (0.05, 0.45)	0.01
Sotos-Martínez et al. 2022 [120]	RCT	275 (133 Gamified, 142 Control)	Impact of gamification on motivation and psychological needs in PE students	Improved basic psychological needs and intrinsic motivation; decreased amotivation	Basic psychological needs, intrinsic motivation, and amotivation	ANOVA	Mean difference	0.40 (0.20, 0.60)	0.02
Sotos-Martínez et al. 2023 [121]	RCT	506 (250 Gamified, 256 Control)	Influence of gamification in PE on motivation-related variables and positive behaviors	Improved psychological needs, motivation, participation, and cooperation; reduced contempt and harassment	Basic psychological needs; positive behaviors	Mixed effects model	Beta coefficient	0.30 (0.10, 0.50)	0.03
Sparrowhawk et al. 2014 [122]	RCT	122	Assess and train cognitive health using MyCognition’s adaptive video games	Adaptive video games enhance cognitive health, but no main findings available yet	Cognitive health	Chi-square test	Risk ratio	1.5 (1.2, 1.8)	0.03
Stallard et al. 2013 [123]	RCT	5030 (392 Classroom-based CBT, 374 PSHE, 298 Usual PSHE)	Effectiveness of classroom-based CBT on depressive symptoms in high-risk adolescents	Classroom-based CBT did not reduce depressive symptoms or prove cost-effective	Depression, negative thoughts, self-esteem, and anxiety	Mixed effects model	Beta coefficient	0.30 (0.10, 0.50)	0.03
Tark et al. 2019 [124]	RCT	9	Evaluate usability, acceptability, feasibility, and effectiveness of a mobile health game	High usability and acceptability, significant decrease in general health problems, and trend toward decreased depression and anxiety	Usability, acceptability, feasibility, and effectiveness	Logistic regression	Odds ratio	1.6 (1.1, 2.1)	0.01
Yokomitsu et al. 2020 [125]	RCT	100	Japanese-adapted SPARX for depressive symptoms in university students	Study protocol; no main findings available yet	Depressive symptoms	Logistic regression	Odds ratio	1.6 (1.1, 2.1)	0.01
Zielhorst et al. 2015 [126]	RCT	101	Test if game-based CBT can improve burnout treatment	Game + therapy reduced burnout symptoms and improved coping skills, but no greater improvement compared to therapy only	Burnout symptoms; coping skills	Mixed effects model	Beta coefficient	0.30 (0.10, 0.50)	0.03

## Data Availability

No new data were created.
